# Real-time measurements of ATP dynamics via ATeams in *Plasmodium falciparum* reveal drug-class-specific response patterns

**DOI:** 10.1128/aac.01690-23

**Published:** 2024-03-19

**Authors:** Eric Springer, Kim C. Heimsch, Stefan Rahlfs, Katja Becker, Jude M. Przyborski

**Affiliations:** 1 Biochemistry and Molecular Biology, Interdisciplinary Research Center, Justus Liebig University, Giessen, Germany; The Children's Hospital of Philadelphia, Philadelphia, Pennsylvania, USA

**Keywords:** *Plasmodium falciparum*, ATP, ATeam1.03YEMK, ATeam1.03-nD/nA, sfpHluorin, 4-aminoquinolines, arylamino alcohols, chloroquine, pyronaridine, amodiaquine, quinine, mefloquine, lumefantrine, plasmodione, SBI-750, methylene blue, cycloheximide, doxycycline, cytosol alkalization, malaria

## Abstract

*Malaria tropica*, caused by the parasite *Plasmodium falciparum* (*P. falciparum*), remains one of the greatest public health burdens for humankind. Due to its pivotal role in parasite survival, the energy metabolism of *P. falciparum* is an interesting target for drug design. To this end, analysis of the central metabolite adenosine triphosphate (ATP) is of great interest. So far, only cell-disruptive or intensiometric ATP assays have been available in this system, with various drawbacks for mechanistic interpretation and partly inconsistent results. To address this, we have established fluorescent probes, based on Förster resonance energy transfer (FRET) and known as ATeam, for use in blood-stage parasites. ATeams are capable of measuring MgATP^2−^ levels in a ratiometric manner, thereby facilitating *in cellulo* measurements of ATP dynamics in real-time using fluorescence microscopy and plate reader detection and overcoming many of the obstacles of established ATP analysis methods. Additionally, we established a superfolder variant of the ratiometric pH sensor pHluorin (sfpHluorin) in *P. falciparum* to monitor pH homeostasis and control for pH fluctuations, which may affect ATeam measurements. We characterized recombinant ATeam and sfpHluorin protein *in vitro* and stably integrated the sensors into the genome of the *P. falciparum* NF54*attB* cell line. Using these new tools, we found distinct sensor response patterns caused by several different drug classes. Arylamino alcohols increased and redox cyclers decreased ATP; doxycycline caused first-cycle cytosol alkalization; and 4-aminoquinolines caused aberrant proteolysis. Our results open up a completely new perspective on drugs’ mode of action, with possible implications for target identification and drug development.

## INTRODUCTION

Malaria is a serious infectious disease and remains one of the greatest public health burdens for humankind. In 2022, an estimated 249 million cases occurred worldwide. Of these, around 608,000 people died, with the majority of victims being children under five. Of *Plasmodium* species, *Plasmodium falciparum* has the highest impact on global health, causing most infections as well as the deadliest form of the disease (1). With growing drug resistance, basic research for the development of new drugs remains critical for global health.

In the asexual blood stages, it was shown, using isotopic labeling (2) and extracellular flux analysis (3), that the parasites rely mainly on glycolysis for adenosine triphosphate (ATP) production and use oxidative phosphorylation to only a minimal extent. Due to this limited metabolic flexibility, glycolytic enzymes (4), as well as upstream processes such as glucose transporters (5), were proposed as potential drug targets. These data also further highlight the importance of ATP as the main energy source for these parasites and stimulate research into ATP metabolism.

There are various approaches for measuring ATP, which are extensively reviewed elsewhere (6). In *P. falciparum*, most ATP measurement approaches to date used luciferase-based bioluminescence assays to measure ATP (7–19) or transgenic luciferase-expressing cell lines as a surrogate for parasite viability (13, 20). However, these approaches have inherent flaws regarding their applicability for mechanistic drug-response interpretation. ATP response toward drug interventions in luciferase-based bioluminescence ATP assays is usually compared to the output of a control group and based on the parasite count, as the exact parasitic cytosolic volume at a given time is hard to define. While using estimations of average parasite volume to calculate a definite [ATP] can partially circumvent this issue, this approach is even less applicable when comparing [ATP] between a control and a drug intervention group in situations in which growth differences are expected, as many drug treatments are known to interfere with protein biosynthesis (21). If a given intervention causes growth retardation and a reduction in cell volume, the intensity of a luciferase-based bioluminescence assay reaction normalized to a certain cell count can decrease in the intervention group, even when the actual molar [ATP] is identical. Additionally, measurements cannot easily be applied to subcellular compartments and are prone to influence from sample preparation. While the intensiometric approach using luciferase-expressing cell lines can be applied to specific subcellular compartments, it also does not necessarily reflect true differences in ATP levels, as the output is responsive to other factors that are expected to change through drug interventions, such as levels of sensor expression or degradation. These difficulties were highlighted by Khan et al. (13), who used both a cell-disruptive luciferase-based bioluminescence assay and a transgenic luciferase-expressing cell line to study ATP-drug response in trophozoite stages. This study showed that while mefloquine and artemisinin caused a clear increase in the amount of ATP over 10 h, the luminescence of the transgenic cell line decreased strongly under the same conditions.

To overcome these limitations, we decided to establish genetically encoded ratiometric FRET-based MgATP^2−^ sensors from the ATeam (Adenosine 5′-Triphosphate indicator based on Epsilon subunit for Analytical Measurements) family in the *P. falciparum* NF54*attB* strain. ATeams were developed by Imamura et al. (22) and offer real-time *in cellulo* measurements of ATP dynamics on a single-cell or even compartmental level. ATeams are composed of a circular permutated mVenus yellow fluorescent protein (YFP) and an mseCFP cyan fluorescent protein (CFP) connected with an ATP-binding domain, the ε-subunit of a *Bacillus subtilis* F_o_F_1_-ATP synthase. Upon ATP binding, this domain changes its conformation, which brings YFP and CFP into close proximity and increases the FRET interaction. Analysis of the YFP- and CFP-specific emission ratio after CFP excitation demonstrates a correlative response toward MgATP^2−^. Thus, this approach should reflect MgATP^2−^ levels independently of the sensor expression level. It should be noted that the physiologically relevant form of ATP is MgATP^2-^; however, we shall follow convention and simply refer to this as ATP, unless otherwise required for clarity.

Modification of the ATP-binding domain led to a series of ATeams with different sensitivities toward ATP (22). Furthermore, red-shifted GO-ATeams were developed based on green fluorescent protein (GFP) and orange fluorescent protein (23). In addition, the dimerization interface of GFP-based proteins was modified to yield sensors with improved dynamic range. Kotera et al. (24) discovered that the L206A mutation yielded the highest dynamic range. We therefore chose the ATeam1.03-nD/nA sensor with an improved dynamic range and sensitivity in the low millimolar range, as well as the ATeam1.03YEMK mutant with a slightly higher sensitivity toward ATP. Despite their advantages over other ATP analysis methods, GFP-based genetically encoded sensors also have pitfalls to consider. Most importantly, the fluorescence of GFP-based proteins may exhibit sensitivity to pH, as reviewed elsewhere (25). Treatment with antiparasitic compounds might induce pH changes in *P. falciparum,* and it has previously been shown that illumination itself can induce a pH drop (26). To control for this, we, in parallel, established a parasite line expressing a genetically encoded pH sensor to monitor possible pH changes caused by drug interventions and sample preparation. pH indicator systems, such as pHluorin (27) or Sypher (28), have already been established in *P. falciparum*. Nevertheless, we chose to establish the superfolder variant developed by Reifenrath and Boles (29). Schuh et al. (30) found that the superfolder mutations appear to cause increased fluorescence intensity in the parasites. Establishing the super folder variant of pHluorin is therefore likely to be advantageous with respect to the increased signal-to-noise ratio.

Another pitfall to consider is the possibility of drug-sensor interactions, which might lead to potential experimental artifacts. We therefore recombinantly produced ATeam and sfpHluorin proteins to analyze drug-sensor interactions *in vitro* and, thereby, control for potential confounding effects.

With these sensors, we aimed to establish a simple and robust platform to analyze ATP-drug response levels and make these tools available for the malaria community. Additionally, we analyzed the ATeam response toward exposure to a selection of different antiparasitic compound classes. These include the 4-aminoquinolines chloroquine (CQ), amodiaquine (AQ), and pyronaridine (PYRO), the arylamino alcohols quinine (QN), mefloquine (MQ), and lumefantrine (LUM), dihydroartemisinin (DHA), the redox-cycler methylene blue [MB (31)], plasmodione [PD (32)], the apicoplast-targeting doxycycline [DOXY (33)], the electron transport chain (ETC)-targeting atovaquone [ATQ (34)], the protein synthesis inhibitor cycloheximide [CHX (35)], as well as the PfGluPho-targeting drug candidate SBI-0797750 [SBI (36)]. With these, we wanted to provoke possibly distinct sensor responses to gain new insights into their mode of action.

## RESULTS

### Recombinant ATeam proteins show a concentration-dependent increase in emission ratio toward ATP

**Fig 1:**
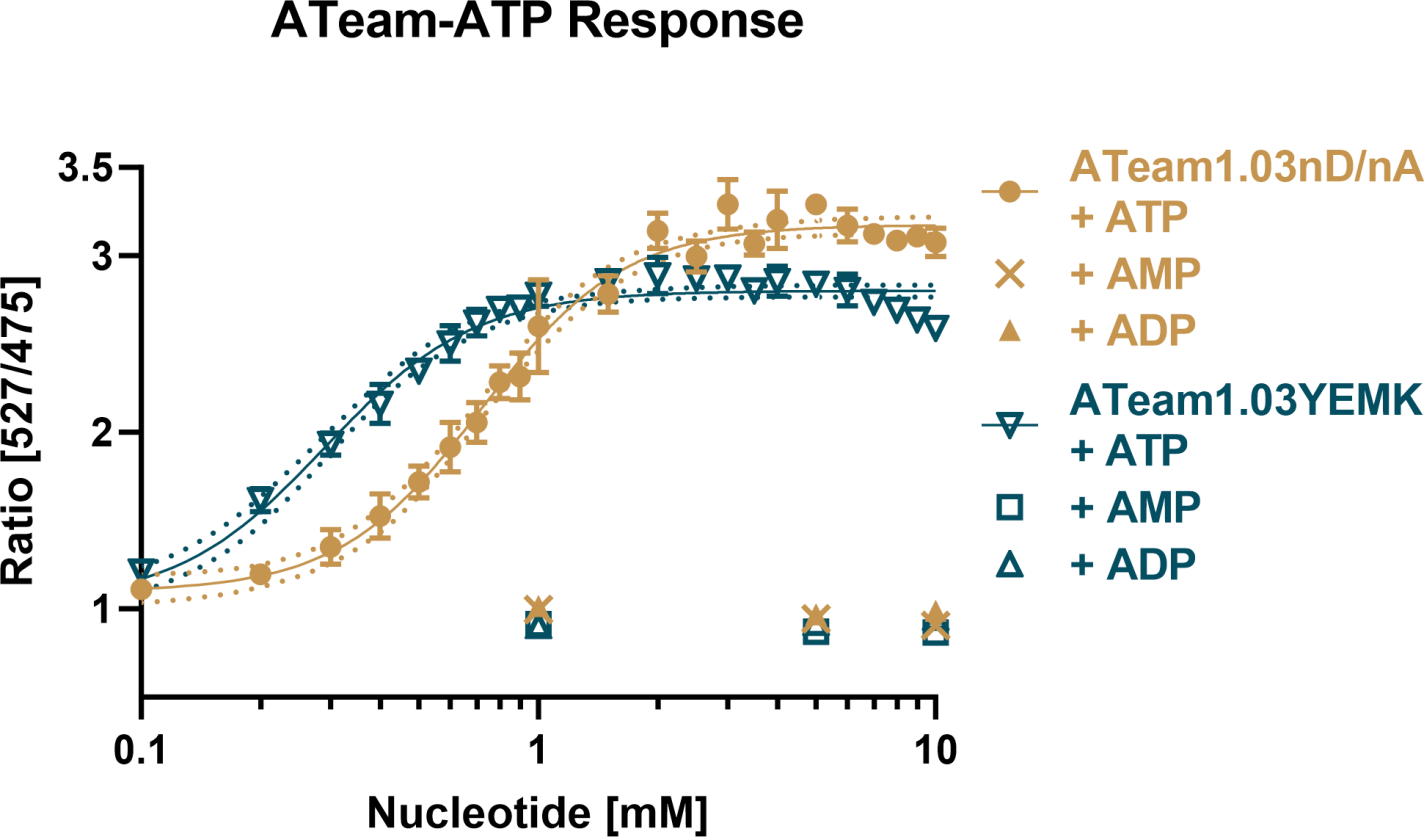
ATeam1.03-nD/nA and ATeam1.03YEMK show a dose-dependent ratio increase in response to ATP. Nucleotides were dissolved in an equimolar solution of MgCl_2_. Measurements were conducted in a plate reader via excitation at 435 nm and emission sensing at 527 nm for YFP and 475 nm for CFP. Mean ratios of *n* = 3 independent experiments are shown. Error bars indicate SD.

To measure potential direct drug-sensor interactions and to characterize ATeam1.03-nD/nA and ATeam1.03YEMK sensors side by side for the first time, we produced His-tagged ATeam proteins recombinantly expressed in *E. coli* and purified them using affinity chromatography. Titration of the ATeam1.03-nD/nA and ATeam1.03YEMK proteins with ATP caused a dose-dependent emission ratio increase, as reported by Kotera et al. (24) and Imamura et al. (22), respectively (Fig. 1). We saw a higher dynamic range for ATeam1.03-nD/nA and a higher sensitivity toward ATP for ATeam1.03YEMK. We also confirmed that neither sensor showed a response to AMP or ADP, as previously shown by De Col et al. (37) and Imamura et al. (22).

**Fig 2:**
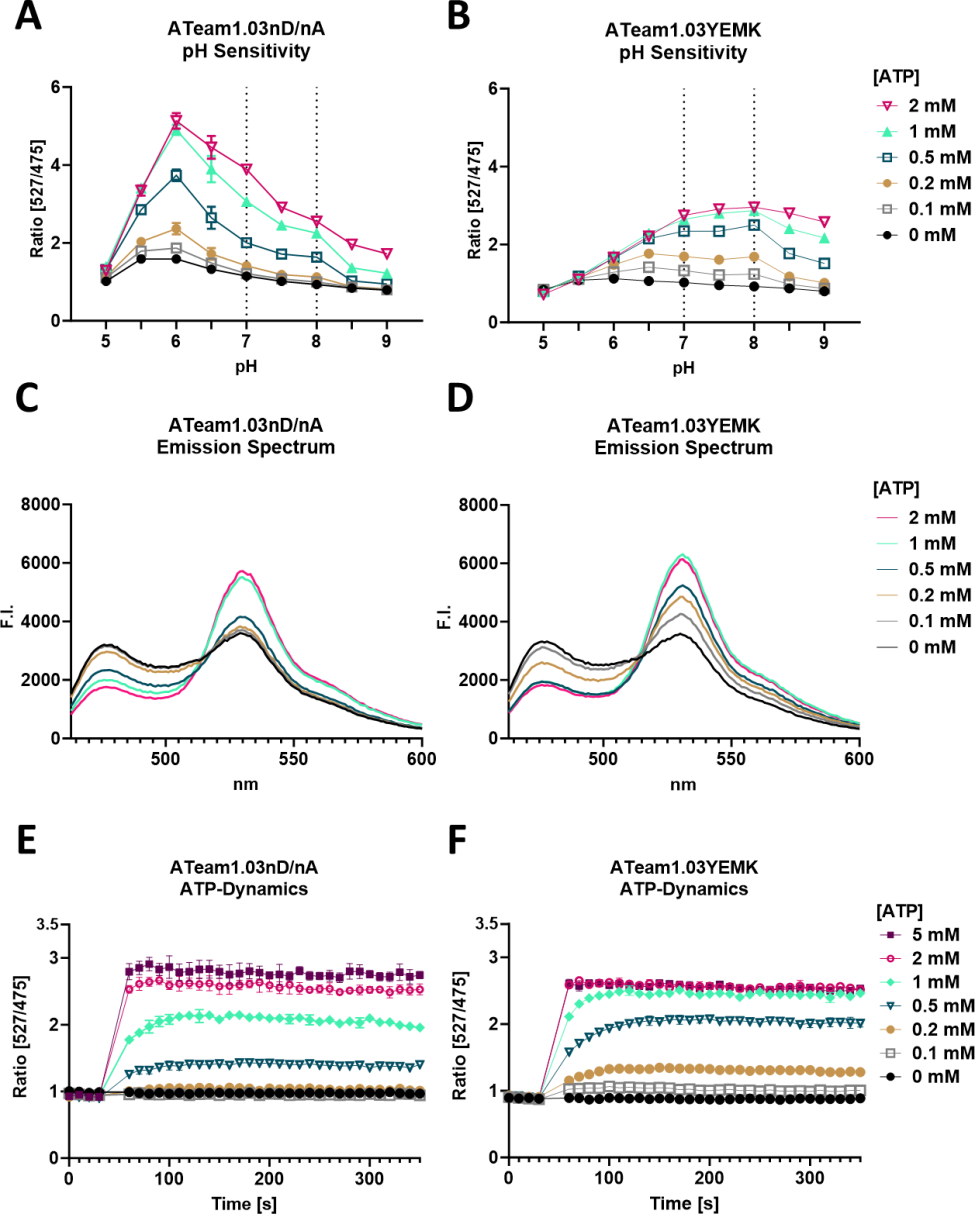
pH, emission spectrum, and time-response characteristics of ATeam1.03-nD/nA and ATeam1.03YEMK. (**A and B**) ATeam1.03-nD/nA shows a higher dynamic range but less pH stability than ATeam1.03YEMK. (**C and D**) Both sensors show a characteristic change in emission spectrum after excitation at 435 nm and emission detection from 463 to 600 nm in response to different [ATP] in accordance with FRET. Error bars were omitted for clarity. (E and F) Both sensors show a rapid response toward changes in [ATP]. The ratio reached a stable level approximately 1 minute after ATP addition. Measurements were conducted via excitation at 435 nm and emission sensing at 527 nm for YFP and 475 nm for CFP. Mean values of *n* = 3 independent experiments are shown. Error bars indicate SD.

We further compared the pH sensitivity, emission spectra, and time responsiveness of both sensors. Despite small discrepancies likely due to different buffer conditions, the ratio response trend is generally in accordance with Imamura et al. (22), Kotera et al. (24), and De Col et al. (37). We saw an apparent increased dynamic range of ATeam1.03-nD/nA toward pH 6 (Fig. 2A). However, the sensor also showed varying ratios at different pH even in the absence of ATP, indicating direct pH sensitivity of the fluorophores. In contrast, the ratio of ATeam1.03YEMK without ATP substrate was consistent between pH 6 and 9 (Fig. 2B). ATeam1.03YEMK showed an apparent lower dynamic range from pH 5 to 7 (Fig. 2B), but the actual FRET response (Fig. S1D) remained consistent. We believe that the decreased ratio between pH 7 and 5 results from an actual lower distribution of MgATP^2−^ species in the lower pH solution, as they are unstable at this pH (38), which was already noted by De Col et al. (37). Both sensors showed typical changes in their emission spectrum in response to increasing [ATP] in accordance with the FRET response, as expected (Fig. 2C and D). With increasing [ATP], CFP fluorescence decreased, while YFP fluorescence increased. Within seconds, both sensors showed a rapid response to changing [ATP] and equilibration within 1 minute (Fig. 2E and F).

ATeam fluorescence and FRET response are influenced by temperature and pH (22). We aimed to imitate the environment in living parasites and therefore chose 37°C and pH 7.34 [cytosolic pH as determined in later experiments (Fig. 9B)] for all experiments with recombinant protein, if not indicated otherwise. Drug-sensor interaction studies did not find any relevant direct interaction between compounds and sensors in our desired concentration range for ATeam1.03YEMK (Fig. S2A) and ATeam1.03-nD/nA (data not shown).

### ATeams can be stably expressed in *P. falciparum* NF54*attB* and are capable of measuring dynamic real-time changes of ATP level in a plate reader format

**Fig 3:**
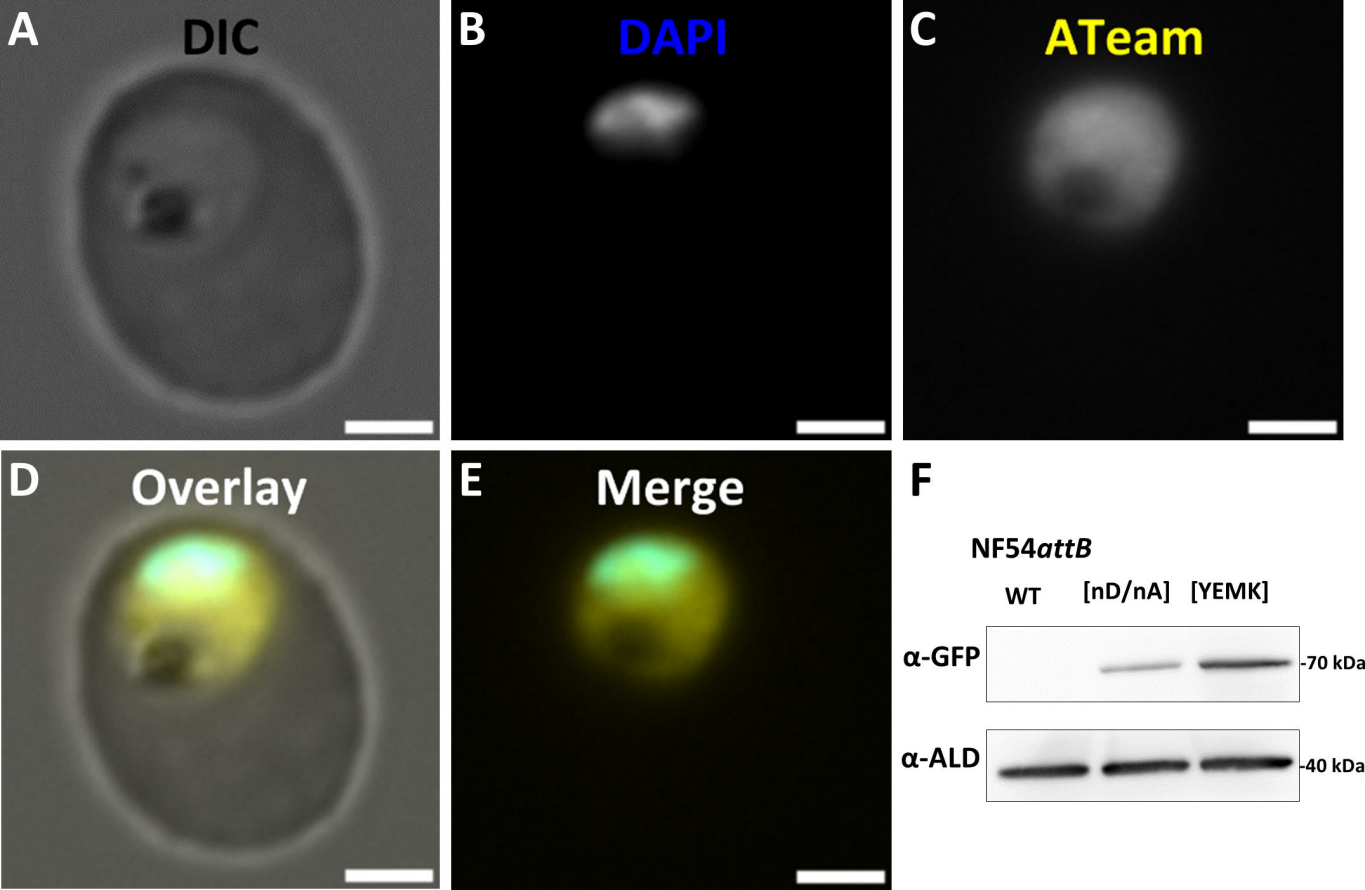
NF54*attB*^[ATeam1.03YEMK]^ and NF54*attB*^[ATeam1.03-nD/nA]^ show cytosolic ATeam fluorescence. DIC (**A**), DAPI (**B**), ATeam (**C**), overlay of panels A–C (**D**), and merge of panels B + C (**E**) of NF54*attB*^[ATeam1.03-nD/nA]^ as representative for both ATeam cell lines. DAPI: ex 353 nm/em 465 nm and ATeam: ex 488 nm/em 509 nm. Scale bar equals 2 µm. (F) Both cell lines show α-GFP signal with the size of ATeam1.03-nD/nA and ATeam1.03YEMK protein (67.8 and 68.0 kDa, respectively). NF54*attB*^[ATeam1.03YEMK]^ shows 1.96 ± 0.38 (SD) fold higher western blot signal than NF54*attB*^[ATeam1.03-nD/nA]^ (*n* = 3) normalized to α-aldolase as loading control.

We used the NF54*attB* cell line lacking a selectable marker (39) to generate our transgenic cell lines. This allows *attB-attP* recombination within the cg6 gene using the mycobacteriophage Bxb1 integrase (40) and, thereby, facilitates rapid generation of stable transfectants. We used limiting dilution to generate clonal lines and PCR for verification (Fig. S3 and S4). NF54*attB*^[ATeam1.03-nD/nA]^ and NF54*attB*^[ATeam1.03YEMK]^ cell lines showed sensor-specific fluorescence in a shape resembling the cytosol of the parasites, as shown for a representative NF54*attB*^[ATeam1.03-nD/nA]^ clonal line (Fig. 3A through E) with similar absolute fluorescence intensity within each cell population (data not shown). Under identical imaging conditions, NF54*attB*^[ATeam1.03YEMK]^ clonal lines showed constantly higher absolute fluorescence intensity than NF54*attB*^[ATeam1.03-nD/nA]^ clonal lines (data not shown). As ATeams are based on CFP and YFP proteins that are derived from GFP, we used cross-reactive anti-GFP antibodies for western blot analyses. In accordance with the lower fluorescence intensity, western blot analysis revealed a higher signal intensity of NF54*attB*^[ATeam1.03YEMK]^ compared to NF54*attB*^[ATeam1.03-nD/nA]^ relative to aldolase loading control, indicating higher sensor abundance in the NF54*attB*^[ATeam1.03YEMK]^ parasites (Fig. 3F).

**Fig 4:**
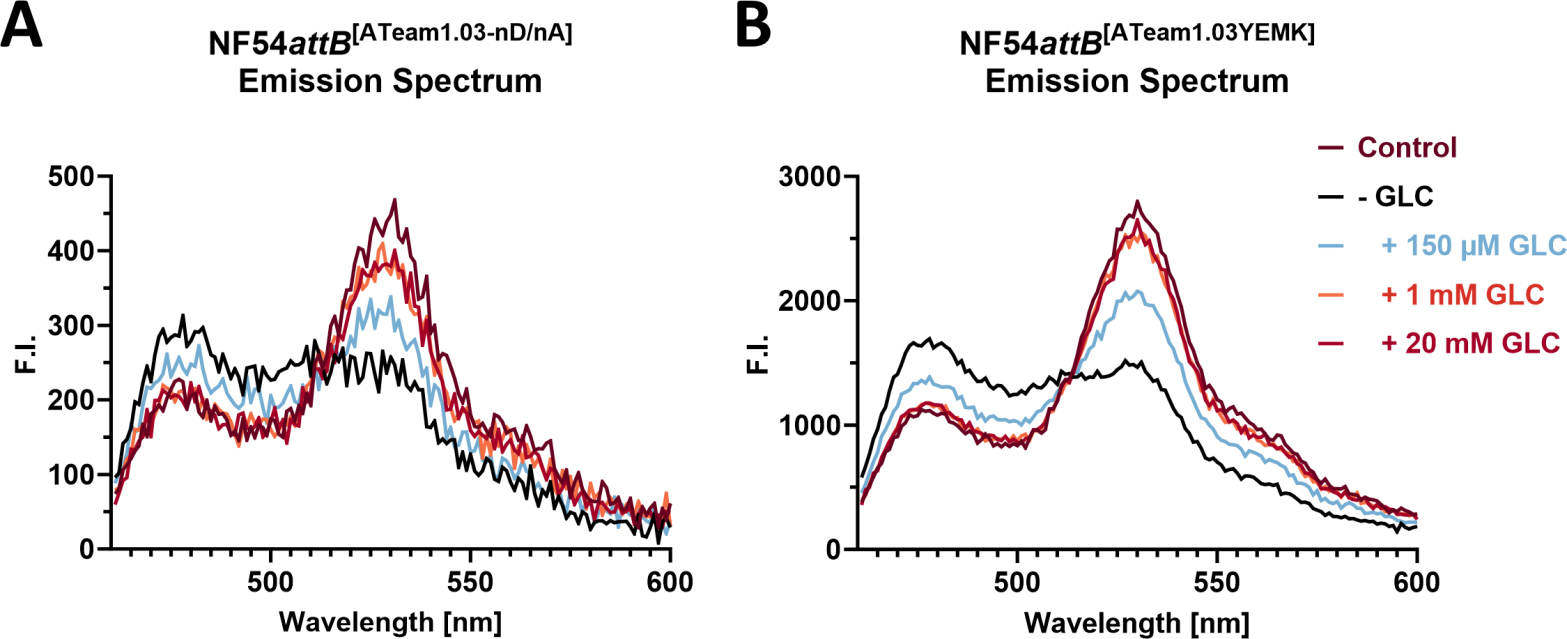
Emission spectrum of NF54*attB*^[ATeam1.03-nD/nA]^ and NF54*attB*^[ATeam1.03YEMK]^ responds to glucose titration in accordance with ATeam FRET. (**A**) NF54*attB*^[ATeam1.03-nD/nA]^ and (**B**) NF54*attB*^[ATeam1.03YEMK]^. Plate reader emission spectra of 2 million magnetic-activated cell sorting (MACS)-enriched trophozoite-infected red blood cells after excitation at 435 nm and emission detection from 463 to 600 nm in response to different [GLC] after 10-minute incubation time are shown. Parasites were washed in phosphate-buffered saline (PBS) for GLC depletion (−GLC) or GLC-rich Ringer’s solution (control). Mean emission spectrum of *n* = 3 independent experiments is shown. Error bars were omitted for clarity.

To demonstrate proof-of-principle of ATeam-expressing cell lines in a plate reader format, we analyzed their emission spectrum after glucose (GLC) starvation in response to the re-addition of GLC. GLC starvation is expected to cause ATP depletion and an according ATeam emission spectrum in the parasites, while GLC re-addition after starvation is expected to restore ATP level, with increasing YFP and decreasing CFP emission peaks. NF54*attB*^[ATeam1.03-nD/nA]^ and NF54*attB*^[ATeam1.03YEMK]^ showed a change in emission spectrum with increasing [GLC] and a 10-minute incubation time, in accordance with the FRET response (Fig. 4A and B). A marked increase in YFP and decrease in CFP peak was already observable with 150 µM GLC. There is no apparent difference between the addition of 1 and 20 mM GLC. No conditions fully replicated the emission spectrum of the control spectrum of parasites that were kept continuously in GLC-rich Ringer’s solution (control). NF54*attB*^[ATeam1.03-nD/nA]^ showed overall lower fluorescence intensity compared to NF54*attB*^[ATeam1.03YEMK]^ under comparable conditions, leading to a lower signal-to-noise ratio.

**Fig 5:**
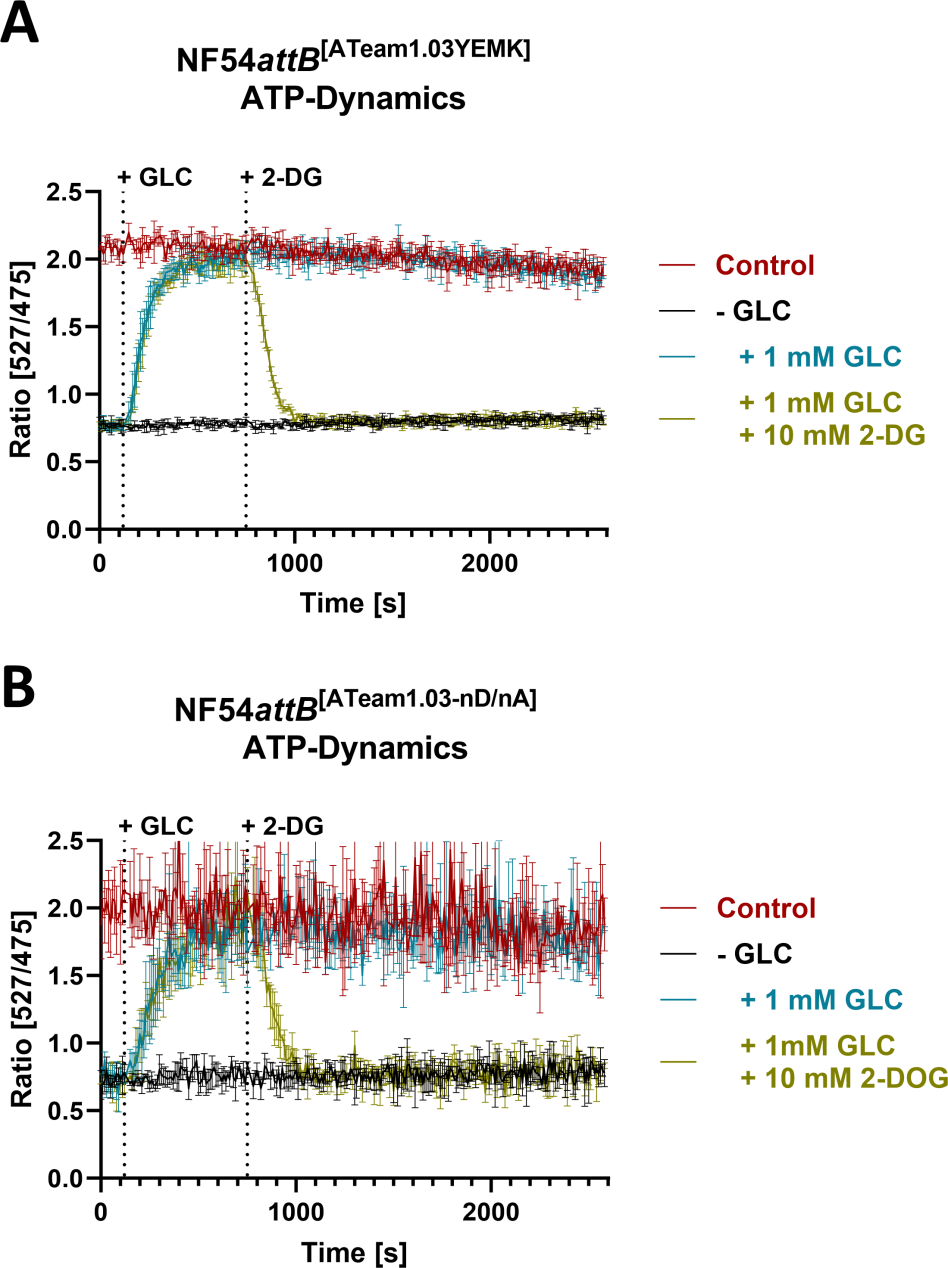
Monitoring of NF54*attB*^[ATeam1.03YEMK]^ and NF54*attB*^[ATeam1.03-nD/nA]^ emission ratio demonstrates dynamic *in cellulo* sensor response in plate reader format. Plate reader emission ratio of 2 million MACS-enriched NF54*attB*^[ATeam1.03YEMK]^ (**A**) or NF54*attB*^[ATeam1.03-nD/nA]^ (**B**) trophozoite-infected red blood cells after excitation at 435 nm and emission sensing at 527 nm for YFP and 475 nm for CFP. Parasites were washed in PBS for GLC depletion (−GLC) or GLC-rich Ringer’s solution (control). Treatment with 1 mM GLC restored the ratio to control in Ringer’s solution. Additional treatment with 10 mM 2-DG after 10 minutes reversed GLC-induced restoration to GLC-deprived baseline ratio. Mean emission ratios of *n* = 3 independent experiments are shown. Error bars indicate SD.

We further demonstrated proof-of-principle of dynamic plate reader measurements over time using treatment with GLC and subsequent addition of the glycolysis inhibitor 2-deoxyglucose [2-DG (41)] after GLC starvation (Fig. 5). GLC addition is expected to restore ATP levels, while the addition of 2-DG is expected to deplete parasite ATP levels. Cells were washed in phosphate-buffered saline (PBS) for GLC depletion or GLC-rich Ringer’s solution as a control. After 2 minutes, cells were treated with either PBS, Ringer’s, or 1 mM GLC solution. Within 10 minutes, GLC-depleted cells treated with 1 mM GLC solution restored their emission ratio almost to that of the control group in Ringer’s solution, indicating restoration of their ATP level. The ratio matched that of the control group in Ringer’s solution over an additional 30 minutes within the plate reader, while both groups showed a small but steady ratio decline over time within the plate reader. In contrast, the addition of 2-DG 10 minutes after recovery of GLC-depleted cells caused a rapid ratio decrease to the baseline level in the GLC-depleted PBS solution. Under the same conditions, NF54*attB*^[ATeam1.03-nD/nA]^ showed a similar response but a much lower signal-to-noise ratio (Fig. 5B). As both total sensor fluorescence and pH independence were lower when using ATeam1.03-nD/nA, we decided to prioritize ATeam1.03YEMK for all further experiments.

### NF54*attB*^[ATeam1.03YEMK]^ can be used to monitor dynamic changes in ATP at single-cell level using fluorescence microscopy

**Fig 6:**
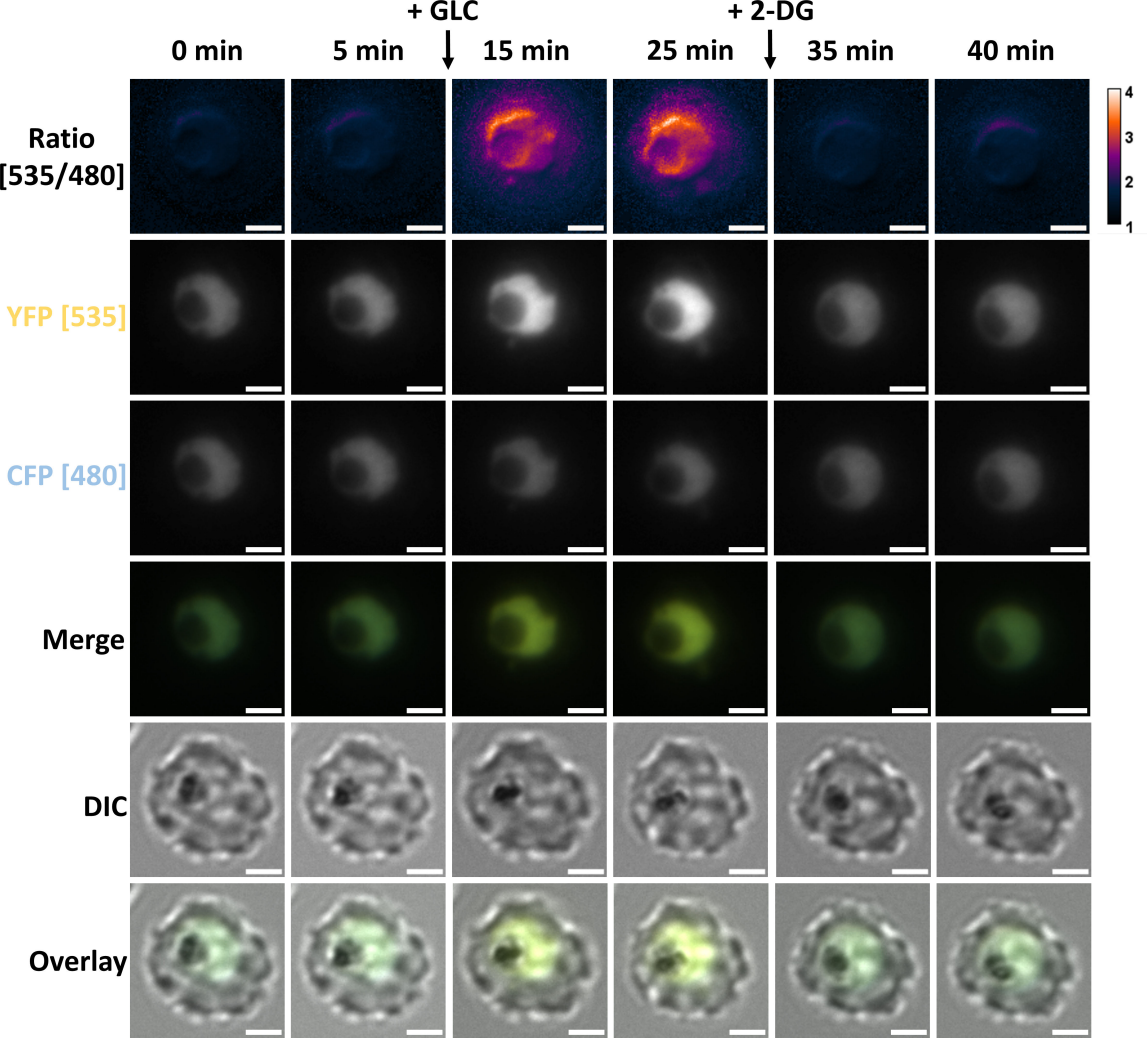
Fluorescence microscopy of NF54*attB*^[ATeam1.03YEMK]^ allows monitoring of ATP-glucose dynamics on a single-cell level. Emission ratio of NF54*attB*^[ATeam1.03YEMK]^ was measured using excitation at 430 nm and emission sensing at 535 and 480 nm for the YFP and CFP channel, respectively. GLC was added to a concentration of 2 mM after taking the 5-minute image. 2-DG was added to a concentration of 10 mM after taking the 25-minute image. Scale bar equals 2 µm.

**Fig 7:**
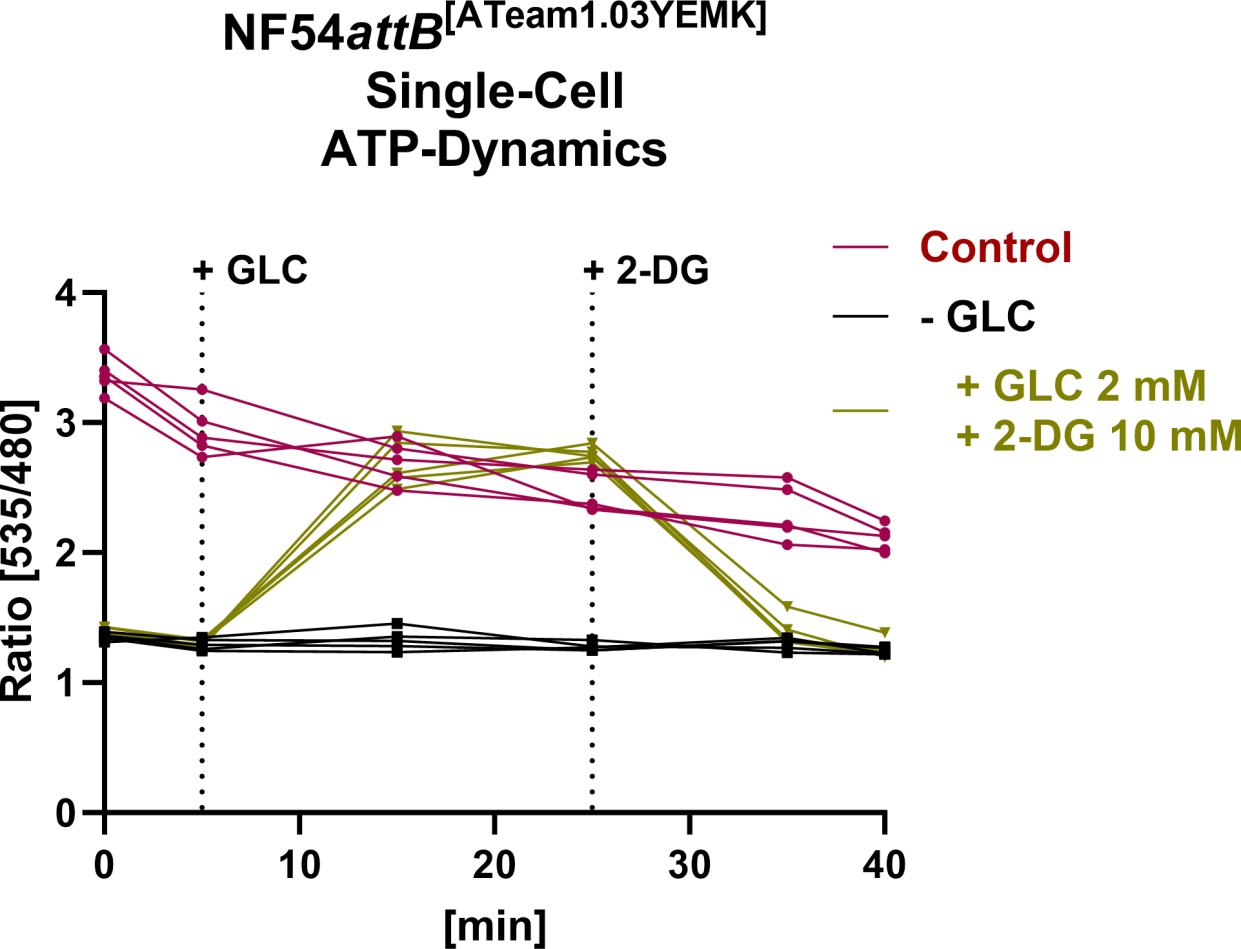
Fluorescence microscopy of NF54*attB*^[ATeam1.03YEMK]^ demonstrates dynamic *in cellulo* sensor response on single-cell level. Emission ratio of NF54*attB*^[ATeam1.03YEMK]^ was measured using excitation at 430 nm and emission sensing at 535 and 480 nm for the YFP and CFP channel, respectively. Background-corrected ratios are shown. Cells were washed and treated either with GLC-rich Ringer’s solution (control) or GLC-depleted PBS (−GLC). Intervention group was treated with GLC and 2-DG after GLC starvation in PBS as indicated. GLC was added to a concentration of 2 mM after taking the 5-minute image. 2-DG was added to a concentration of 10 mM after taking the 25-minute image. Continuous measurements of *n* = 5 parasites/group are shown.

Following on from plate reader measurements, we established NF54*attB*^[ATeam1.03YEMK]^ measurements on a single-cell level using fluorescence microscopy. Similar to the plate reader experiments (Fig. 5), we treated GLC-starved parasites with GLC and subsequent addition of 2-DG. Figure 6 shows the emission ratio, overlay, YFP-, and CFP-emission channel of a representative trophozoite-infected red blood cell (iRBC) over time. The cell dynamically reacts to GLC addition with an increase and decrease of fluorescence intensities in the YFP and CFP channels, respectively, leading to an increase in emission ratio. The process is reversed via the application of 2-DG. Plotting the emission ratio of multiple single cells over time demonstrates a dynamic *in cellulo* sensor reaction in a fluorescence microscope (Fig. 7). The measurements also reveal a steady emission ratio decrease in untreated control parasites that may reflect limited photobleaching.

### The pH sensor sfpHluorin allows robust measurement of cytosolic pH and is capable of measuring dynamic pH changes in a plate reader

**Fig 8:**
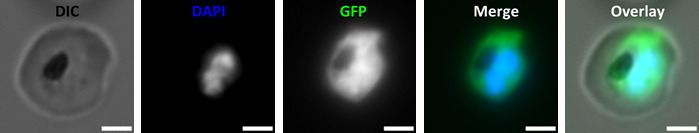
NF54*attB*^[sfpHluorin]^ shows cytosolic GFP fluorescence. DIC, DAPI, GFP, merge, and overlay of a representative trophozoite stage parasite of NF54*attB*^[sfpHluorin]^ are shown. DAPI: ex 353 nm/em 465 nm and GFP: ex 488 nm/em 509 nm. Error bar equals 2 µm.

As shown in Fig. 2, *in cellulo* ATeam measurements could be affected by pH fluctuations. To control for this, we generated parasites expressing the genetically encoded pH sensor sfpHluorin. Additionally, we produced His-tagged sfpHluorin protein to characterize the sensor’s behavior *in vitro* and to control for possible direct drug-sensor interactions. pH titration and changes in emission and excitation spectra in a plate reader (Fig. S5) were in accordance with Reifenrath and Boles (29). We could not detect any direct drug-sensor interactions in our desired concentration range (Fig. S2B). We generated stable sfpHluorin-expressing cell lines and used limiting dilution to isolate clonal lines. To verify stable integration, we used PCR (Fig. S6). The NF54*attB*^[sfpHluorin]^ clonal line showed sensor-specific fluorescence in a shape resembling the parasitic cytosol (Fig. 8).

**Fig 9:**
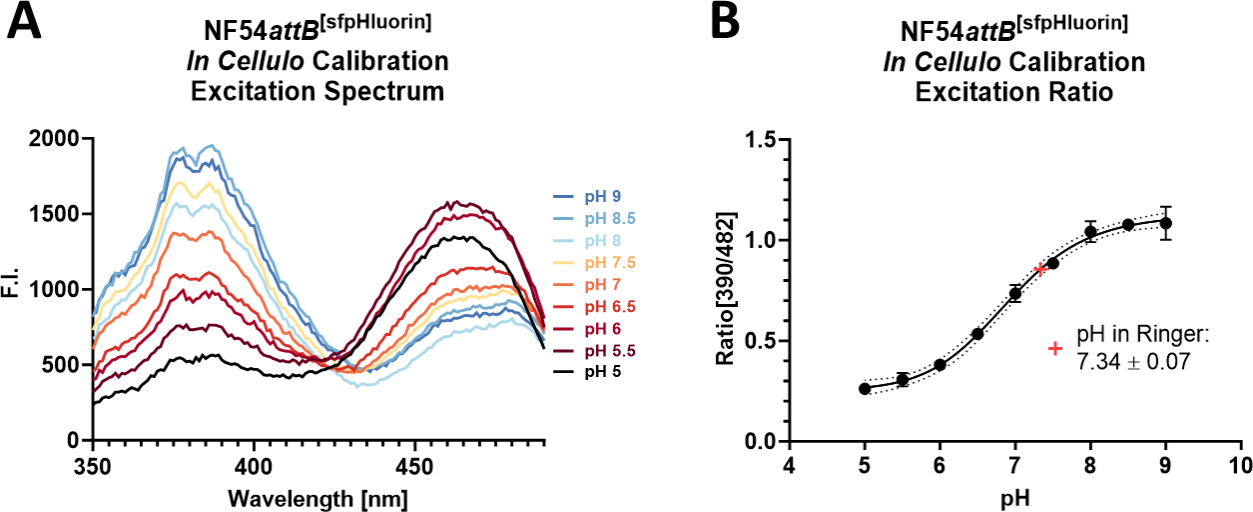
NF54*attB*^[sfpHluorin]^ *in cellulo* calibration in plate reader format. A total of 1 million MACS-enriched trophozoite-iRBCs were incubated with 10 µM nigericin for 30 minutes at 37°C in 384-well plates in buffers with pH from 5 to 9. (A) Excitation spectra from 350 to 490 nm with emission sensing at 530 nm show pH-responsive peaks around 390 and 482 nm. Error bars were omitted for clarity. (B) Fitting a calibration curve using anexcitation ratio of 390–482 nm with emission sensing at 530 nm of nigericin-treated cells in pH buffers allows pH determination of untreated cells in Ringer’s solution. Means of *n* = 3 independent experiments are shown. Error bars indicate SD.

To demonstrate proof-of-principle of NF54*attB*^[sfpHluorin]^ *in cellulo*, we applied nigericin pH calibration. Nigericin is an ionophore, allowing pH equilibration of the cytosol with the extracellular media (42). Spectral analysis of nigericin-permeabilized NF54*attB*^[sfpHluorin]^-iRBCs reveals excitation spectra changing in response to pH in accordance with that of recombinant sfpHluorin, with pH-responding peaks around 390 and 482 nm (Fig. 9A). The excitation ratio of 390–482 nm plotted over pH level allows the fitting of a sigmoidal calibration curve for *in cellulo* pH measurements with a high dynamic range in the physiological relevant range of the parasitic cytosol between pH 6 and 8. Estimation of parasite resting pH in physiologic Ringer’s solution determines a pH ± SD of 7.34 ± 0.07 (Fig. 9B), in large agreement with previous determinations, such as 7.29 ± 0.01 (11), 7.31 ± 0.02 (43), or 7.3 ± 0.05 (26).

**Fig 10:**
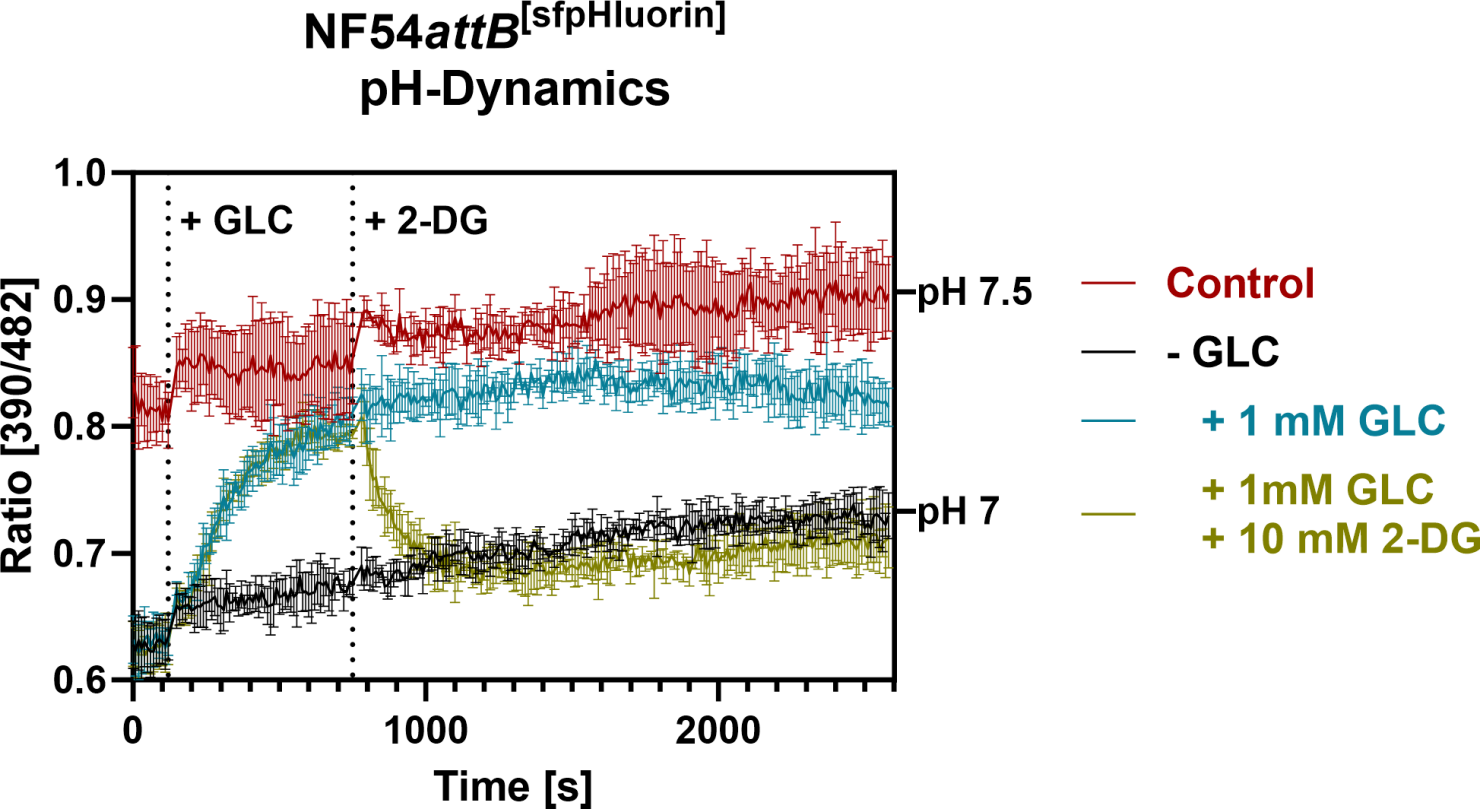
Monitoring of NF54*attB*^[sfpHluorin]^ excitation ratio demonstrates dynamic *in cellulo* sensor response in plate reader format. Plate reader excitation ratio of 1 million MACS-enriched trophozoite infected-iRBCs after excitation at 390–482 nm with emission sensing at 530 nm. Parasites were washed in PBS for GLC depletion (−GLC) or GLC-rich Ringer’s solution (control). Treatment with 1 mM GLC restored the ratio close to control in Ringer’s solution. Additional treatment with 10 mM 2-DG after 10 minutes reversed GLC-induced restoration close to the GLC-starved baseline ratio. Mean emission ratios of *n* = 3 independent experiments are shown. Error bars indicate SD.

To further demonstrate the suitability of NF54*attB*^[sfpHluorin]^ for measuring dynamic pH changes, the cell line underwent GLC starvation, recovery, and subsequent 2-DG treatment as in previous experiments (Fig. 10). Starvation from GLC in PBS led to a lower excitation ratio compared to the control. Treatment with 1 mM GLC restored the excitation ratio to almost that of the control. Subsequent 2-DG treatment after 10 minutes rapidly reversed the GLC-induced recovery. The dynamic pH response of the parasite in response to GLC starvation and recovery shown here matches the findings of Saliba and Kirk (11), who showed that starvation from GLC causes a drop in parasite cytosolic pH and that recovery from acidification is heavily impaired under GLC starvation.

### NF54*attB*^[sfpHluorin]^ can be used to monitor pH changes at a single-cell level using live-cell imaging

**Fig 11:**
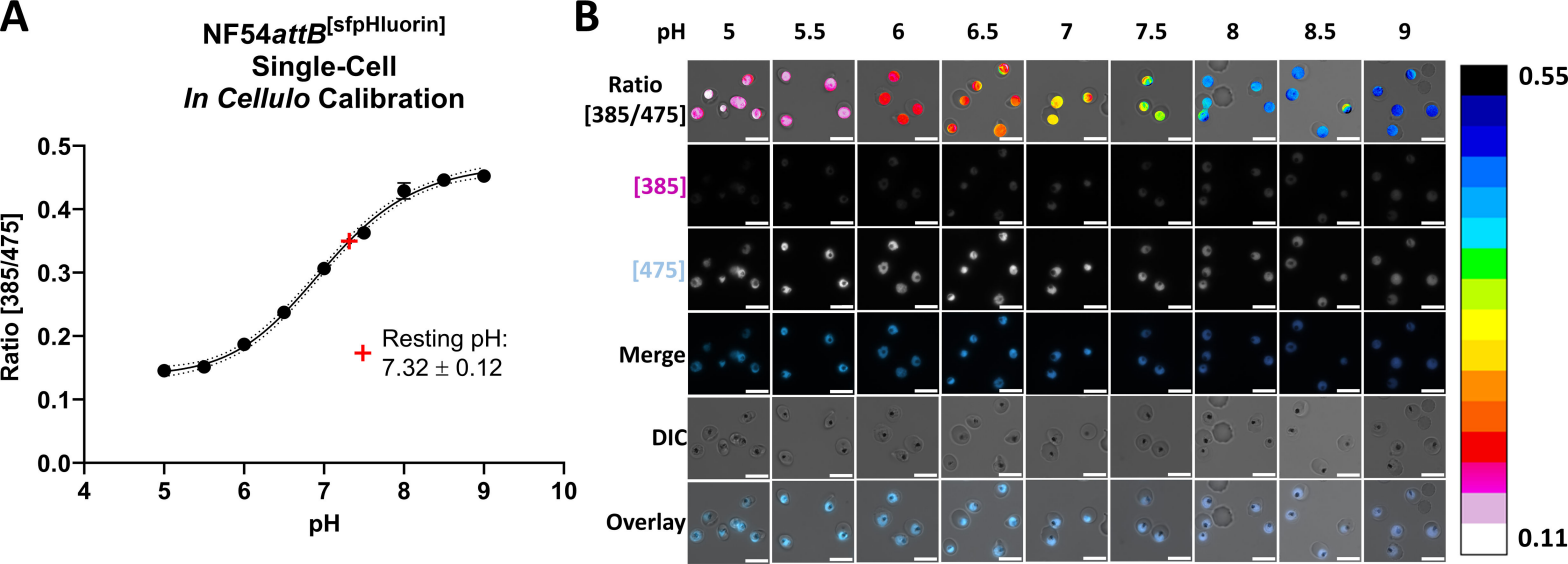
NF54*attB*^[sfpHluorin]^ single-cell *in cellulo* calibration in epifluorescence microscope. MACS-enriched trophozoite-iRBCs incubated with 10 µM nigericin for 30 minutes at 37°C in buffers with pH from 5 to 9 show a pH-responsive increase in 385–475 nm excitation ratio at 525 nm. (A) Fitting a calibration curve allows resting pH determination of untreated cells in Ringer’s solution. Measurements show the mean of *n* = 3 independent experiments with 100 cells ± SD. (B) Representative single-cell images of calibration experiments. Calibration bar color codes pixel by pixel 385–475 nm excitation ratio at 525 nm. Scale bar equals 10 µm.

To control pH changes that might occur during sample preparation and measurements in the single-cell microscopy setup for NF54*attB*^[ATeam1.03YEMK]^, we further established NF54*attB*^[sfpHluorin]^ measurements in a similar live-cell setup. We used the nigericin calibration method as described earlier. Accordingly, we observed a 385–475 nm excitation ratio increase with increasing pH and a high dynamic range between pH 6 and 8. Through interpolation of a standard curve, we could determine the resting pH (mean ± SD) of parasites in the microscopy setup in Ringer’s solution to be 7.32 ± 0.12 (Fig. 11A), in strong accordance with our measurements made using a plate reader (7.34 ± 0.07, Fig. 9B). Additionally, we demonstrate morphologic integrity and cytosolic fluorescence localization of single iRBCs after nigericin calibration (Fig. 11B).

### NF54*attB*^[ATeam1.03YEMK]^ shows distinct response patterns toward different drug classes

Having established NF54*attB*^[ATeam1.03YEMK]^ and NF54*attB*^[sfpHluorin]^ as ATP and pH measurement systems, we wished to use these new tools to study parasite responses to different established drug classes and promising drug candidates. We incubated the sensor cell lines with the compounds at approximately 100× and 10× of their EC_50_ concentrations (Table S1) to induce rapidly detectable effects over an incubation time of 4 and 6 h. Exceptions to this approach were DOXY and the model inhibitor of protein synthesis CHX. DOXY was used at the fixed concentrations of 5 and 1 µM to account for first- and second-cycle effects (33), and CHX was used at a single dose of 50 µg/mL to demonstrate the effect of a protein synthesis inhibitor.

To detect the ATeam response, we used the microscopic measurement approach with *n* = 5 independent experiments and calculated the mean of 100 parasites for each independent experiment with a concurrent vehicle-treated control group in each microscopic dish. We used this randomized block design to tease out parasite responses to different compound classes and used two-way ANOVA for statistical analysis. To cope with the multiple comparisons problem, we used the false discovery rate [FDR (44)], as this approach retains high power for the identification of drug response patterns. Discoveries with 5% FDR are reported with a single asterisk. Further statistical analysis can be found in Tables S2 to S7.

**Fig 12:**
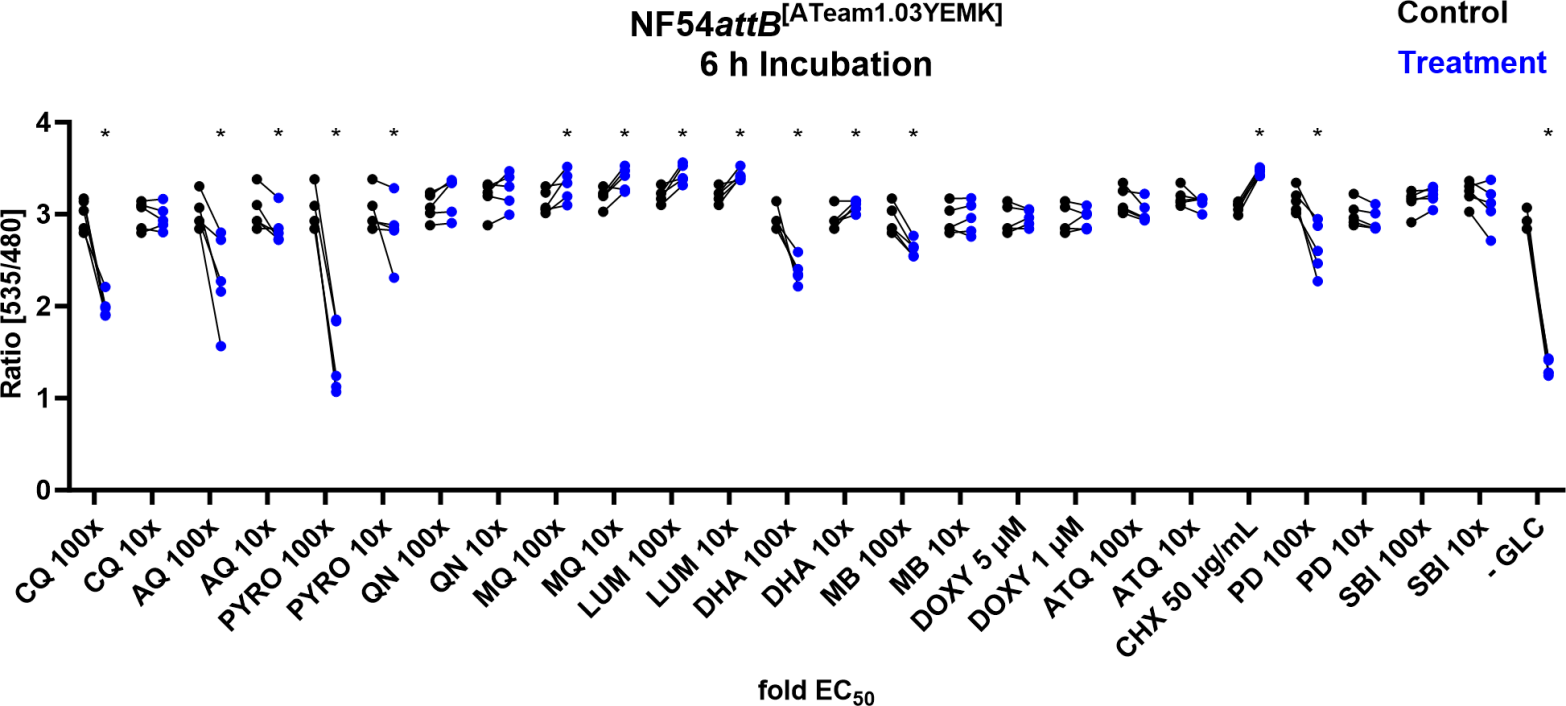
NF54*attB*^[ATeam1.03YEMK]^ shows distinct ratio changes in response toward different drug classes after 6-h incubation. Fluorescence microscopic measurement of MACS-enriched trophozoite-iRBCs. Each measurement corresponds to the 535–480 nm emission ratio after excitation at 430 nm. Means of 100 single-cell analyses of compound intervention (treatment) and concurrent vehicle control (control) with *n* = 5 independent experiments are shown. Treatment groups included CQ, AQ, PYRO, QN, MQ, LUM, DHA, MB, DOXY, ATQ, CHX, PD, SBI, and −GLC. Cells were starved from GLC through washing in PBS just before measurement. If not indicated otherwise, compounds were applied with 100× and 10× EC_50_ concentrations. Asterisks indicate discovery with FDR = 5%.

After 6-h incubation, we found distinct ATeam response toward different drug classes (Fig. 12). The 4-aminoquinoline compounds CQ, AQ, and PYRO caused a marked drop in ATeam emission ratio at 100× of their EC_50_ and, except for CQ, additionally at 10× EC_50_. In contrast, the arylamino alcohols MQ and LUM caused an increase in emission ratio, both at 100× and 10× EC_50_ concentration. The structurally related QN did not show any effect on the ATeam emission ratio. DHA caused a mixed effect on the emission ratio. We could detect a decrease in emission ratio for 100× EC_50_, while we could detect an increase for 10× EC_50_. The redox-active compounds MB (31) and PD (32) caused an emission ratio decrease at 100× EC_50_ but not at 10× EC_50_. The apicoplast-affecting drug (33), DOXY, the inhibitor of the mitochondrial ETC (34), ATQ, as well as the PfGluPho inhibitor, SBI-0797750 (36), did not show any effects under the given conditions. Additionally, we included the effects of GLC starvation (−GLC) and the inhibitor of protein biosynthesis CHX in our study. As expected, GLC starvation just before measurement caused a drastic drop in the emission ratio. In contrast, CHX caused an increase in ratio. Overall, we found similar results for all tested compounds already after 4-h incubation. However, the results were less pronounced and not detected as discovery according to the FDR for 10× EC_50_ of AQ, PYRO, MQ, DHA, SBI, and 100× and 10× EC_50_ of LUM (Fig. S7).

**Fig 13:**
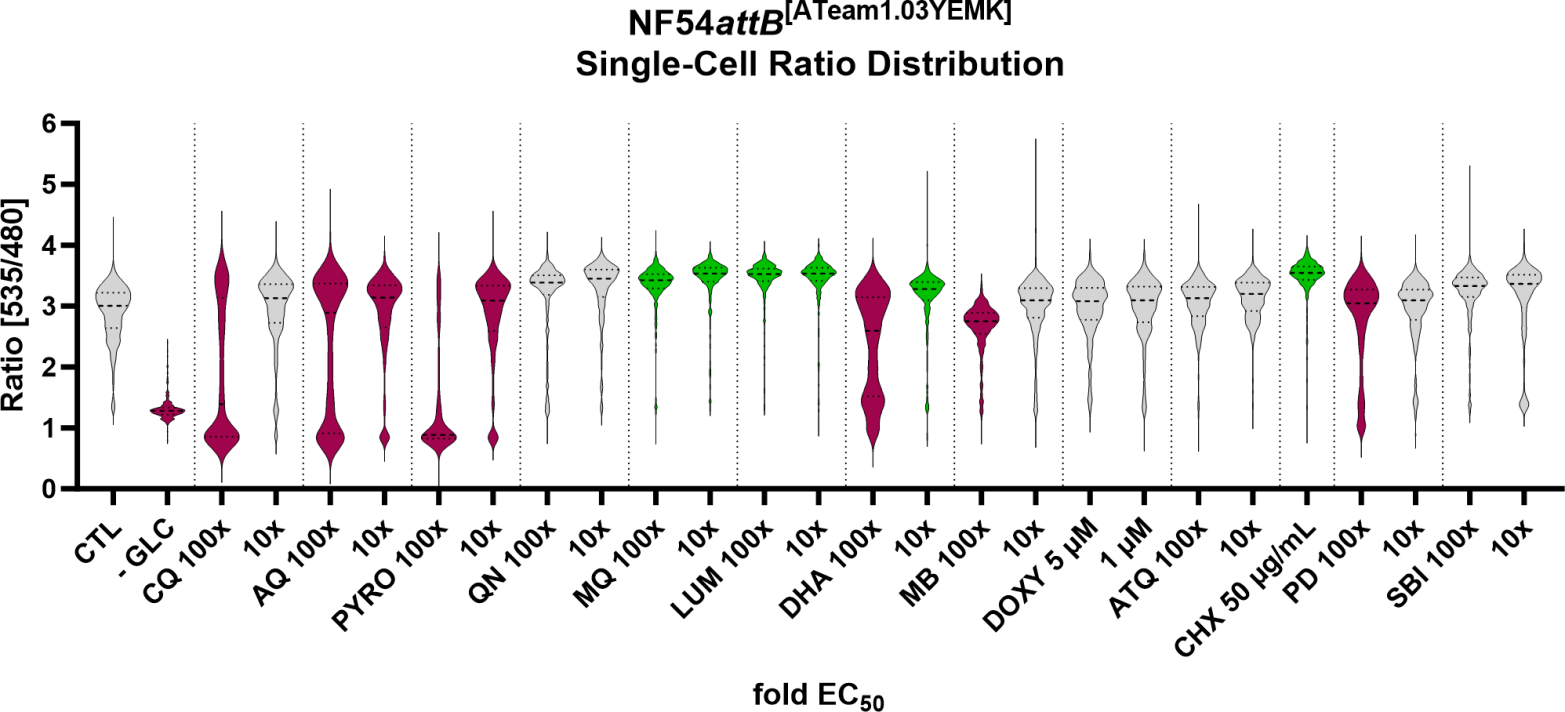
NF54*attB*^[ATeam1.03YEMK]^ shows distinct single-cell emission ratio distribution in response toward different drug classes. Fluorescence microscopic measurement of MACS-enriched trophozoite-iRBCs. Each measurement corresponds to the 535–480 nm emission ratio after excitation at 430 nm. Pool of *n* = 5 independent experiments with each 100 single-cell analyses is shown. Treatment groups included CQ, AQ, PYRO, QN, MQ, LUM, DHA, MB, DOXY, ATQ, CHX, PD, SBI, and −GLC. Cells were starved from GLC through washing in PBS just before measurement. If not indicated otherwise, compounds were applied with 100× and 10× EC_50_ concentrations. Colors indicate up- (green) or down (magenta)-shifted discoveries according to the statistical analysis described in Fig. 12.

Pooling the single-cell data of the *n* = 5 independent experiments gives further insights into the compound-induced ATeam response. Though not suitable for statistical analysis, as the pooling and lack of concurrent control would make statistical analysis questionable, we nevertheless observed distinct ATeam response patterns toward different drug classes on a single-cell level (Fig. 13). The 4-aminoquinolines, CQ, AQ, and PYRO, caused a distinctive bimodal ratio distribution at 100× EC_50_ concentration. A notable proportion of their ratio distributions are located below 1, while even cells starved of GLC did not show such low ratios. In contrast, the distributions of the arylamino alcohols, QN, MQ, LUM, in addition to CHX, and SBI are more compressed and higher in location compared to a representative control group. DHA caused mixed effects in the ratio distribution. It showed a compressed and higher-located distribution at 10× EC_50_, while it shows a tendency toward a bimodal and downwardly skewed distribution at 100× EC_50_. MB showed a tendency for compression and lower ratios at 100× EC_50_. PD showed a tendency toward a lower and bimodal distribution. DOXY and ATQ did not show any apparent change in their distributions compared to the representative control.

### pH changes induced by drug interventions are generally small and unlikely to affect *in cellulo* ATeam measurements

**Fig 14:**
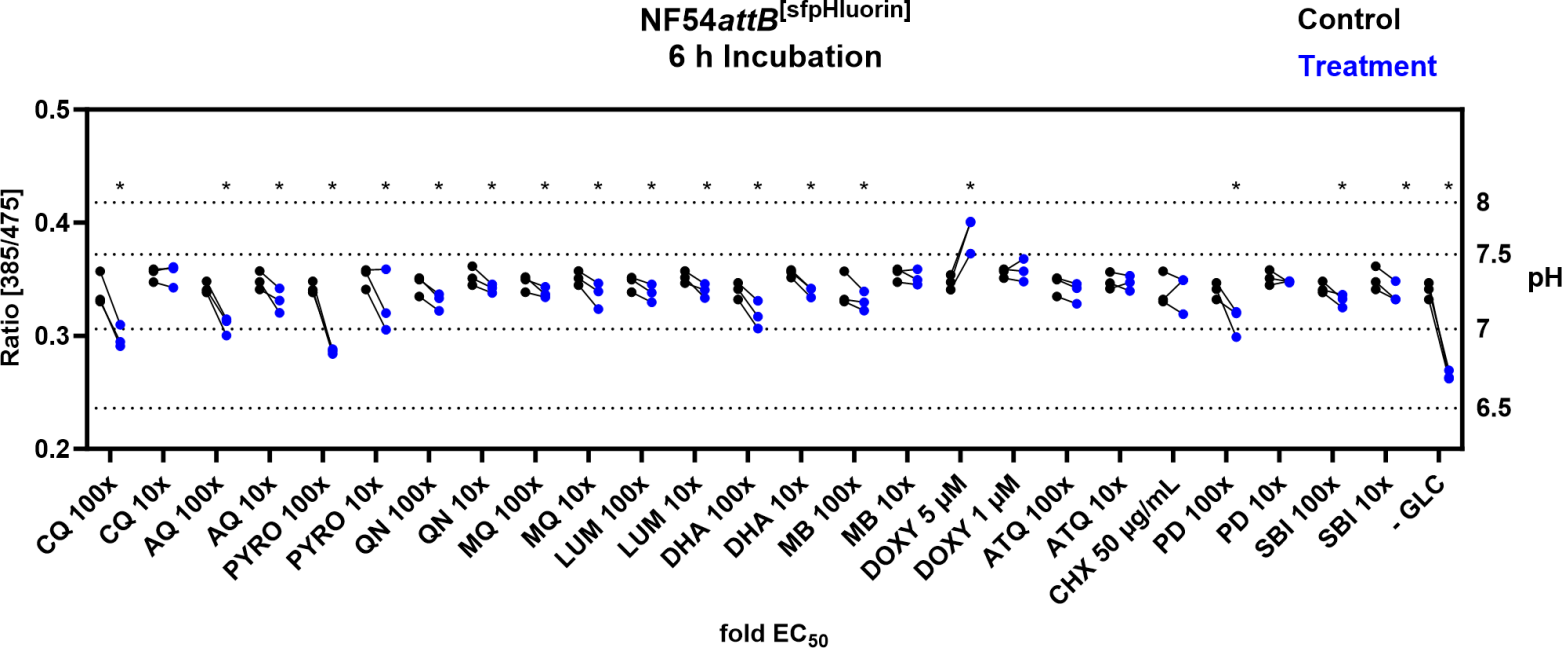
NF54*attB*^[sfpHluorin]^ shows predominantly marginal ratio changes in response toward different drug classes after 6-h incubation time. Fluorescence microscopic measurement of MACS-enriched trophozoite-iRBCs. Each measurement corresponds to the 385–475 nm excitation ratio at 525 nm emission. Means of 100 single-cell analyses of compound intervention (treatment) and concurrent vehicle control (control) with *n* = 3 independent experiments are shown. Treatment groups included CQ, AQ, PYRO, QN, MQ, LUM, DHA, MB, DOXY, ATQ, CHX, PD, SBI, and −GLC. Cells were starved from GLC through washing in PBS just before measurement. If not indicated otherwise, compounds were applied with 100× and 10× EC_50_ concentrations. Asterisks indicate discovery with FDR = 5%.

As shown in Fig. 2B, ATeam1.03YEMK showed a stable ratio response within the pH range of 6–8. In order to investigate if pH changes might influence ATeam measurements upon treatment, all compound treatments shown in Fig. 12 were repeated with NF54*attB*^[sfpHluorin]^. We used the microscopic measurement approach with *n* = 3 independent experiments and calculated the mean of 100 parasites for each independent experiment with a concurrent vehicle-treated control group in each microscopic dish. The pH levels were interpolated from excitation ratio measurements using the *in cellulo* calibration shown in Fig. 11. After 6-h incubation, we found distinct sfpHluorin responses toward different drug classes (Fig. 14). The 4-aminoquinoline compounds, CQ, AQ, PYRO, as well as PD at their 100× EC_50_ concentration caused a marked drop below pH 7. Among all compounds tested, only DOXY at a concentration of 5 µM caused an increased pH; however, this is still in the range classed as reliable for ATeam measurements. All other compounds caused no or only marginal sfpHluorin excitation ratio changes through the concentrations tested. In addition to that, GLC starvation just before measurement caused the strongest excitation ratio drop, indicating a mean pH ± SD of 6.71 ± 0.02. With the exception of CQ and LUM 10× EC_50_ concentrations, we could detect the same significant trends after only 4-h incubation time (Fig. S8). Mean pH with a 95% confidence interval for all treatments after 4- and 6-h incubation is listed in Tables S9 and S8, respectively.

As previously demonstrated, ATeam1.03YEMK protein shows a stable ratio response between pH 6 and 8, indicating the pH stability of the fluorophores in that range. Although all measured average values lie within this range, plotting the pooled ratio distribution of *n* = 3 independent experiments with each containing 100 single-cell analyses to the interpolated pH reveals that some proportion of cells exhibit drastic changes in pH levels, which is masked when analyzing only mean ratios (Fig. S9). For example, at the higher DOXY concentration (5 µM), a number of single-cell measurements lie outside of the calibration range.

### V**arious compounds cause reduced parasite size**

**Fig 15:**
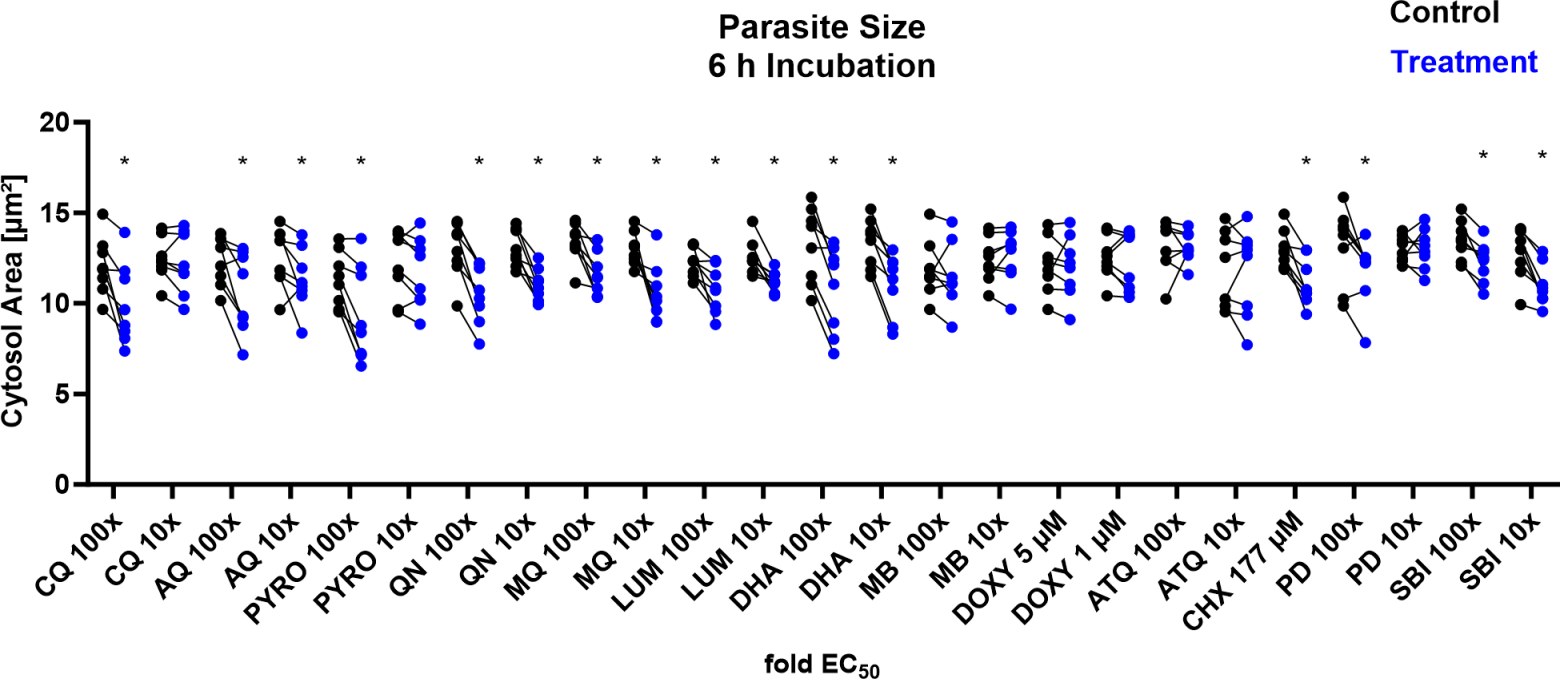
NF54*attB*^[ATeam1.03YEMK]^ and NF54*attB*^[sfpHluorin]^ show decreased parasite size in response toward different drug classes after 6-h incubation. The mean cytosol size of 100 MACS-enriched trophozoite-iRBCs derived from automated ROI definition from fluorescence microscopic measurements of NF54*attB*^[ATeam1.03YEMK]^ (*n* = 5) and NF54*attB*^[sfpHluorin]^ (*n* = 3) is combined. Analyses of compound intervention (treatment) and concurrent vehicle control (control) with *n* = 8 independent experiments are shown. Treatment groups included CQ, AQ, PYRO, QN, MQ, LUM, DHA, MB, DOXY, ATQ, CHX, PD, and SBI. If not indicated otherwise, compounds were applied with 100× and 10× EC_50_ concentrations. Asterisks indicate discovery with FDR = 5%.

The single-cell image analysis for the NF54*attB*^[ATeam1.03YEMK]^ and NF54*attB*^[sfpHluorin]^ drug response measurements included the definition of region of interest (ROI) that corresponds to the area of the parasites. To gain a deeper understanding of the parasite’s response to the compound interventions, we analyzed the mean sizes of these ROIs as a proxy for parasite size and compared them to the mean of the control group (Fig. 15). We found that the 4-aminoquinoline compounds, CQ, AQ, and PYRO, caused a decreased parasite size at their 100× EC_50_ concentrations. Additionally, PYRO caused decreased size already at 10× EC_50_. The arylamino alcohols, QN, MQ, and LUM, in addition to DHA, and SBI caused a decreased cytosol size at both 100× and 10× EC_50_. In addition, 100× EC_50_ of PD and 50 µg/mL CHX caused reduced size. MB, DOXY, and ATQ did not show an effect on parasite size with any of the concentrations tested. Most of the effects were already detected after 4-h incubation. Only AQ 10×, LUM 100×, LUM 10×, and SBI 100× EC_50_ did not show a detectable effect after this shorter incubation time (Fig. S10).

### Parallel analysis of drug responses reveals common patterns

**Fig 16:**
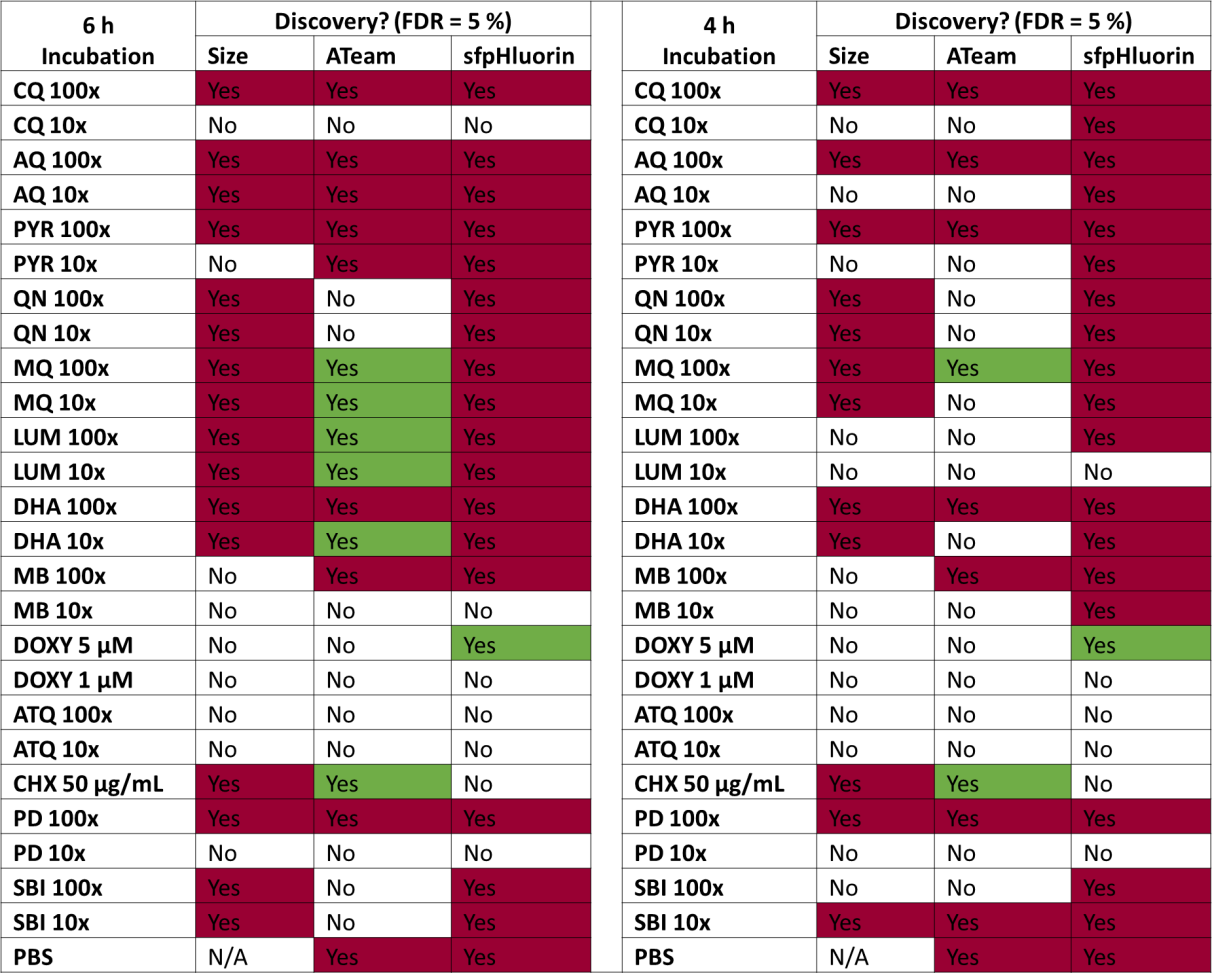
Heatmap of sensor and parasite size drug-response patterns shows coinciding effects of compound treatments. The table shows a synopsis of the effects of different compound treatments on size, ATeam emission ratio, and sfpHluorin excitation ratio of NF54*attB*^[ATeam1.03YEMK]^ and NF54*attB*^[sfpHluorin]^ with concentrations in multiples of EC_50_ or otherwise indicated concentrations after 4- or 6-h treatment time. Effects are classified as discovery (Yes), or no discovery (No) with an FDR of 5%. Color indicates if treatment caused an increase (green) or decrease (magenta) in group means, in case a treatment was classified as discovery in analyses described in Fig. 12, 14, and 15; Fig. S9 to S11. Treatment groups included CQ, AQ, PYRO, QN, MQ, LUM, DHA, MB, DOXY, ATQ, CHX, PD, and SBI.

The characteristics we analyzed are not truly independent, as severe metabolic alterations that affect ATP or pH levels are likely to also cause growth effects and vice versa. Accordingly, examination of cytosol size and sensor response patterns in the synopsis shows that most of the compounds tested caused either an effect on none or all of the analyzed characteristics after 6-h incubation time (Fig. 16). CQ 10×, MB 10×, ATQ 100× and 10×, and PD 10× EC_50_, as well as DOXY 1 µM did not show any effects classified as discovery. CQ 100×, AQ 100× and 10×, PYRO 100×, MQ 100× and 10×, LUM 100× and 10×, DHA 100× and 10×, and PD 100× caused effects on all characteristics. PYRO 10× and MB 10× caused effects only on ATeam ratio and pH, while QN 100× and 10× and SBI 100× and 10× only caused effects on size and pH. CHX only caused effects on cytosol size and ATeam ratio, while DOXY 5 µM caused only an increased pH. Measurements after 4-h incubation showed similar trends, though for some characteristics less pronounced and not detected as discovery.

### Mefloquine but not amodiaquine prevents chloroquine-mediated ATeam and sfpHluorin ratio depletion

**Fig 17:**
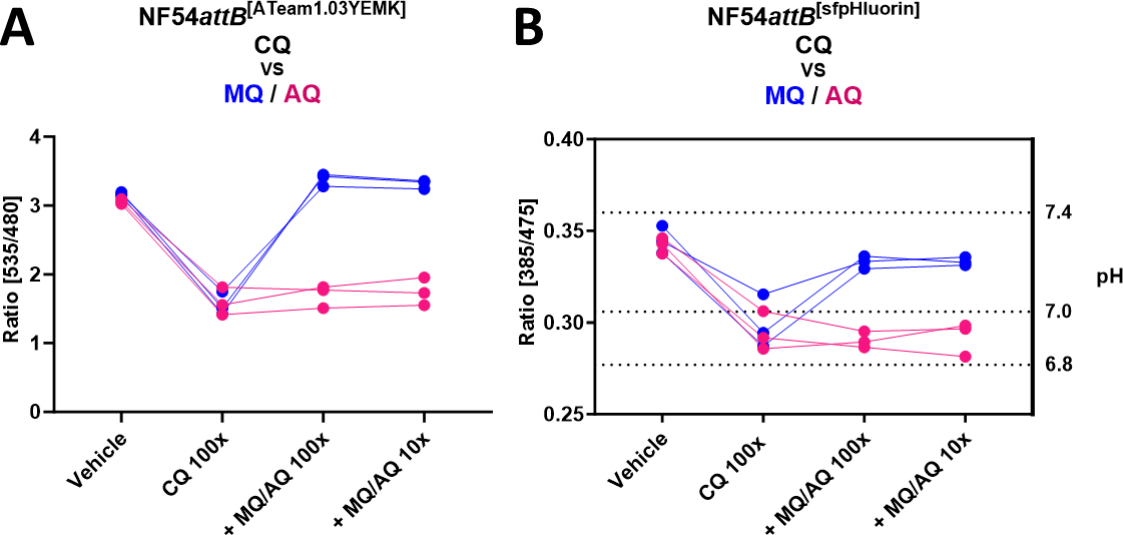
MQ but not AQ prevents CQ-mediated ATeam and sfpHluorin ratio depletion. Fluorescence microscopic measurement of MACS-enriched NF54*attB*^[ATeam1.03YEMK]^ (**A**) and NF54*attB*^[sfpHluorin]^ (**B**) trophozoite-iRBCs. Each measurement corresponds to the 535–480 nm emission ratio after excitation at 430 nm, or 385–475 nm excitation ratio with emission sensing at 525 nm, as indicated. Means of 100 single-cell analyses after incubation with multiples of EC_50_ with *n* = 3 independent experiments are shown. NF54*attB*^[ATeam1.03YEMK]^ and NF54*attB*^[sfpHluorin]^ were treated either with vehicle control, 100× CQ, 100× CQ and 100× MQ, 100× CQ and 10× MQ (blue) or with vehicle, 100× CQ, 100× CQ and 100× AG, and 100× CQ and 10× AQ (magenta) of their EC_50_ concentration for 6 h.

We could detect a characteristic drop in ATeam ratio and pH (Fig. 16) caused by the 4-aminoquinolines, CQ, AQ, and PYRO, after incubation with 100× EC_50_ for 6 h. CQ, AQ, and PYRO are thought to kill the parasites through the inhibition of hemozoin formation with the accumulation of free heme (45). The detected effects on the sensor ratios might therefore be heme mediated. To investigate further the specificity of these effects, we analyzed the interaction of CQ, as a representative of the 4-aminoquinoline group, with MQ or AQ. MQ is reported to inhibit CQ-mediated heme accumulation (46). Therefore, we predicted a preventative effect of MQ on the CQ-mediated ATeam ratio and pH drop. AQ, which belongs to the 4-aminoquinoline group, as does CQ, was used as a control in this setting. We found that, indeed, MQ but not AQ prevented CQ-mediated ATeam1.03YEMK (Fig. 17A) and sfpHluorin (Fig. 17B) ratio depletion at 100× and 10× of their EC_50_ concentrations. In contrast, AQ did not show such a preventative effect.

### Mefloquine but not amodiaquine prevents chloroquine mediated ATeam sensor degradation

**Fig 18:**
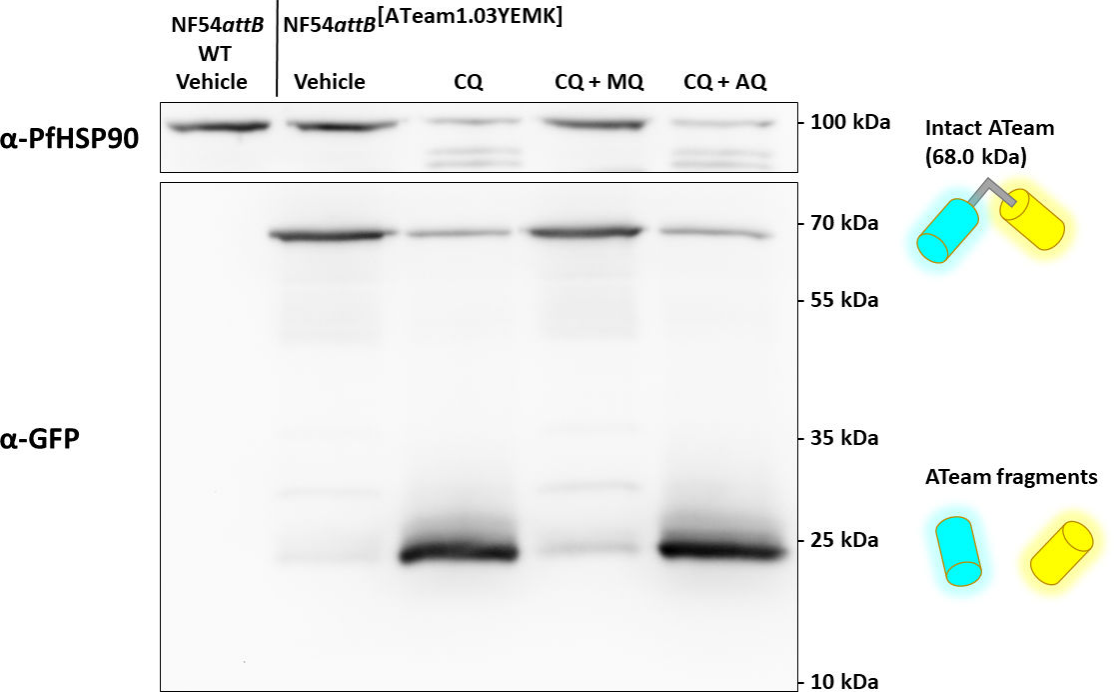
α-GFP western blot of NF54*attB*^[ATeam1.03YEMK]^ demonstrates CQ-induced sensor degradation and the protective effect of MQ. α-GFP western blot of 5 million NF54*attB* (WT) or NF54*attB*^[ATeam1.03YEMK]^ trophozoite-iRBCs treated with CQ, CQ and MQ, CQ and AQ, or vehicle control at 100× EC_50_ for 6 h each.

As shown in Fig. 13, we see a distinct emission ratio response of NF54*attB*^[ATeam1.03YEMK]^ toward 4-aminoquinoline compounds. The single-cell ratio distributions resemble a bimodal distribution with ratio allocations way below the ratio allocations of parasites starved from GLC. As the ATeam sensors are based on intramolecular FRET, such low ratios are only expected if the sensors are degraded, or, if the cytosolic pH is heavily affected. We found only moderate effects on cytosolic pH (Fig. 14). Therefore, we were interested in whether CQ treatment led to ATeam sensor degradation and whether MQ could prevent such degradation. Thus, we carried out western blot analysis of cells treated with CQ, CQ and MQ, CQ and AQ, or vehicle control (Fig. 18). In mock-treated NF54*attB*^[ATeam1.03YEMK]^ parasites, we could detect a clear band around 70 kDa, at the predicted size of ATeam1.03YEMK protein (68.0 kDa). Treatment with 100× EC_50_ CQ for 6 h caused a considerably lower band intensity at this size, but a concomitant detection of a band at around 25 kDa. This band is at the molecular weight of the protease-resistant core of GFP variants, thus likely indicating proteolytic cleavage of the ATeam sensor. Parasites treated with both CQ and MQ did not, or only to a marginal extent, show such a band; however, simultaneous treatment with both CQ and the 4-aminoquinoline AQ still resulted in the degradation product.

We detected PfHSP90 as a loading control. We could detect a similar PfHSP90 signal around the 100-kDa marker band in the CQ + MQ group and in the untreated control group. In the CQ and CQ + AQ-treated group, we could only detect a faint signal at this molecular mass and two additional bands between 70 and 100 kDa, indicating HSP90 degradation products. As housekeeping genes, such as HSP90, can be up- or downregulated as a stress response or degraded under proteolytic stress, we demonstrate via Ponceau S total protein staining that the western blot signal distribution is unlikely to be explained only by unequal sample loading (Fig. S11).

## DISCUSSION

In this report, we largely verified previous *in vitro* data for ATeam proteins (22, 24, 37), with small variations likely being due to slightly different experimental conditions, such as temperature or buffer composition. Overall, all sensors exhibited spectral properties as expected and were assessed by us as functional. We further excluded direct compound-sensor interactions as a cause of ratio changes, under the conditions studied. Our analyses did, however, identify a pH dependence for ATeam1.03-nD/nA under specific conditions, which must be considered when applying such sensors, especially under conditions, such as drug treatments, which may cause pH shifts. Following this, we established ATeam1.03YEMK and ATeam1.03-nD/nA measurements in *P. falciparum*. We could successfully demonstrate dynamic changes in ATP levels, both in a plate reader, as well as in live single-cell fluorescence microscopy. Both sensor cell lines exhibit spectral properties as expected and changed their emission ratio in response to GLC starvation, recovery, and subsequent 2-DG treatment in accordance with the expected corresponding ATP levels. We therefore assess our measurement systems as validated. NF54*attB*^[ATeam1.03-nD/nA]^ showed a marked lower fluorescence intensity in the parasites and an unstable pH response of the recombinant protein and was thus excluded from further analyses.

For our ATP-drug-response analyses, we are interested in qualitative changes in ATP level, not absolute quantitative measurements of [ATP]. Instead, we present the ATeam system as a reliable semi-quantitative tool for measuring ATP responses in *P. falciparum*. Based on our *in vitro* and *in cellulo* characterization, we are certain about NF54*attB*^[ATeam1.03YEMK]^’s capabilities to measure exact cytosolic MgATP^2−^ level, even though it is difficult or impossible to extrapolate absolute molar [ATP] from our data. While it might be tempting to use *in vitro* characterization of the recombinant sensor protein for calibration to deduce to quantitative *in cellulo* [ATP], we think that the deduction from ratios of recombinant sensor protein to intracellular [ATP] is neither viable nor, in our case, necessary. We cannot exclude that the exact intracellular milieu has an effect on quantitative ATP measurements using our sensor, and our calibration experiments, while attempting to mirror the most important conditions, cannot account for many other (unknown) factors. To avoid this issue, various *in situ* calibration protocols have been attempted in different cell systems; however, these revealed discrepancies between the values obtained using different methods, making it impossible to define a “gold standard” (47, 48). We wish to note that both parasites and the host cell generate ATP from GLC, and ATP starvation is likely to also block ATP production in the host cell. The exchange (and direction) of ATP between parasite and host cell is a complex and poorly understood topic (7, 49, 50). We cannot formally exclude an extra-parasite source for some cytosolic ATP but believe that this is not relevant to our current measurements.

As we observed the pH dependence of the sensor under certain conditions, we established the genetically encoded pH sensor sfpHlourin to monitor pH changes under the experimental conditions we applied. Our determinations for the resting pH ± SD of 7.34 ± 0.07 and 7.32 ± 0.12 in our plate reader and live single-cell measurement setup, respectively, are in large agreement with previously reported methods (11, 26, 43) with larger discrepancies likely due to the differences in experimental conditions (26, 27, 51, 52). We, therefore, assess our sfpHluorin measurement system and our interpolated pH determinations as validated.

Having established our experimental system, we then analyzed parasite responses to compounds from varying chemical classes. For our experimental setup, we used 100× and 10× EC_50_ compound concentrations to induce rapid effects in a relatively short time frame, which have been determined in previous studies to largely reflect at least the peak concentration of most of the drugs *in vivo* (53–61). For setting up our drug-response assays, we used starvation from GLC as a control for total ATP depletion. Thus, ratios observed below this level would be indicative of other non-specific effects such as sensor degradation. We observed the highest ratios when treating parasites with CHX. CHX is an inhibitor of protein synthesis, and as protein synthesis is the major ATP-consuming process in rapidly proliferating microorganisms (62), its inhibition has been observed to lead to increases in cytosolic ATP concentration in other systems (63). Our data suggest that, following CHX treatment, ATP concentrations approach sensor saturation, indicated by the compression of ratio distribution values.

Taken individually, each parameter we measured does not allow strong conclusions to be drawn about differing modes of action. However, taken as a whole and encompassing measurement of ATP, pH, and cell size, our results reveal distinct response patterns to 4-aminoquinolines, arylamino alcohols, artemisinins, a selection of redox cyclers, as well as of DOXY, ATQ, and CHX.

Like the 4-aminoquinolines presented in this study, the arylamino alcohols QN, MQ, and LUM all inhibit the formation of hemozoin *in cellulo* and increase free heme to a limited extent (64). However, they also show distinct differences in their cellular effects and resistance mechanisms, as reviewed before (65). As resistances against CQ and MQ are inversely correlated, it is now consensus that the mode of action of arylamino alcohols, such as MQ, is distinct to that of 4-aminoquinolines and that their target most likely lies in the parasite cytosol rather than in the food vacuole (66). In line with this, MQ counteracts many effects of CQ such as Hb accumulation (46), and QN inhibits CQ-induced heme accumulation (67). Aside from this, and even after decades of research, the exact mechanism of action of MQ and QN is still uncertain and hotly debated (21, 68, 69), and for LUM, the situation is even less clear. QN, MQ, and LUM all seem to share a downstream inhibitory effect at 10× EC_50_ on protein synthesis (21) although the exact point at which they exert their influence is unknown. Our study found increasing ATeam ratios after MQ and LUM treatment with 100× and 10× EC_50_. We found a similar trend for QN regarding its single-cell ratio distribution. These findings support Khan et al. (13), who detected increased ATP levels in response to 5× EC_50_ MQ. Additionally, for all three drugs, we measured a reduced parasite size that could be indicative of translation inhibition. As already described for CHX, inhibition of protein synthesis seems to elevate the ATP level in *P. falciparum*. Thus, the increased ATP level following MQ or LUM treatment might be the consequence of such translational arrest.

For artemisinin (ART)-based drugs, their modes of action are thought to be even more pleiotropic in nature than for the other compound classes. Recently, Ward et al. (66) reviewed artemisinin’s mode of action. Upon activation via heme iron, ART is activated to a free radical leading to the alkylation of heme, proteins, lipids, DNA, and other biomolecules and thereby, among others, interfering with Hb metabolism and inducing proteotoxic stress. In our study, we found that 10× EC_50_ of the artemisinin derivative DHA leads to an increased ATP level, while 100× EC_50_ leads to a decreased ATP level. Considering the large number of potential targets and biochemical pathways influenced by DHA treatment, we do not believe that it is possible to draw strong conclusions as to the exact mechanism leading to these ATP responses.

In our selection of compounds, we included two redox cyclers, PD and MB. PD is a 1,4-naphthoquinone derivative, active against all asexual stages, and a promising lead compound (32). In contrast, MB is the first synthetic chemotherapeutic that was developed. The history, redox-cycling activity, and other pleiotropic targets of MB were reviewed extensively by Schirmer et al. (31). In our study, we found that both compounds exert quite similar effects, causing a moderate drop in ATeam ratio and only a slight drop in cytosolic pH. PD treatment of parasites under similar conditions to those we applied has been shown to lead to a significant shift in the cytosolic redox potential toward oxidation (70). Kehr et al. (71) showed that the proteome of *P. falciparum* is highly redox regulated, including glycolytic enzymes such as PfGAPDH. One explanation of our results is that, faced with oxidative stress, the parasites upregulate the activity of the pentose phosphate pathway at the cost of glycolysis.

DOXY and other tetracyclines are known to target the apicoplast’s ribosomal translation and kill the parasites with a delayed death phenotype (33) resulting largely from the inhibition of isoprenoid synthesis and subsequent loss in the second cycle of the apicoplast itself (72). At higher concentrations (up to 10 µM), the first cycle effect was also noted, which could be negated by supplementation with either the isoprenoid precursor IPP or iron, suggesting that this effect is apicoplast dependent (73). Interestingly, we find that 5 µM DOXY causes an increase in cytosolic pH, while this did not occur upon the addition of 1 µM, indicating that first cycle effects include misregulation of cytosolic pH by a mechanism as yet unknown.

SBI-0797750 is a promising drug candidate with nanomolar activity targeting PfGluPho, the bi-functional fusion protein of glucose 6-phosphate dehydrogenase and 6-phosphogluconolactonase, and treatment of parasites at 1× EC_50_ led to a significant shift in the cytosolic redox potential toward oxidation after 4 h (36). As PfGluPho redirects glucose-6-phosphate from glycolysis to the oxidative pentose phosphate pathway, inhibition of this enzyme may be expected to allow a higher rate of glycolysis and thus higher rate of ATP production. However, we could not detect dramatic effects on the ATeam ratio, even though the single-cell ratio distribution does appear to be more compressed and elevated compared to control parasites. One explanation is that the more oxidative environment generated upon SBI treatment leads, in parallel, to downregulation of glycolytic activity as noted above. Alternatively, changes in ATP levels over the course of the experiment may be too small to be evident.

ATQ is an inhibitor of the ETC (34). Malaria parasites rely, during the blood stages, largely on energy production via glycolysis (2, 3), and the only essential function of the ETC during these stages appears to be regeneration of ubiquinone for pyrimidine synthesis (74). In accordance with this, mitochondrial inhibitors are reported to cause only slight alterations in cellular ATP levels over a short time frame (9). In our experimental setup, ATQ was the only compound that did not show any effects on the trophozoite stages after 4 or 6 h at any of the concentrations tested. Our data fit the observations of Wilson et al. (75) that ATQ asserts its effects only on later-stage parasites.

It is a general consensus that the 4-aminoquinolines CQ, AQ, and PYRO kill the parasites through inhibition of hemozoin formation and the induction of similar metabolomics effects (76). However, they also show differences regarding the size of their effect on the distribution of heme species (46, 64, 77). In our study, we found that all of the 4-aminoquinolines included in our study show similar distinct sensor responses. High concentrations of 100× EC_50_ led to reduced parasite size, a moderate drop in pH to 7 or slightly below 7, as well as ATeam ratio depletion, which was indicative of a drop in [ATP]. Unusually, the single-cell ATeam ratio distribution dropped below the expected minimum ratio defined by parasites starved from GLC. One explanation for this behavior would be sensor degradation. To investigate this phenomenon, we used CQ as a representative of the compound class and monitored sensor integrity via western blot. Indeed, treatment of parasites with CQ led to sensor degradation. In addition to effects on ATeam sensor integrity, we also observed degradation of the PfHSP90 loading control under 100× EC_50_ CQ. Interestingly, co-treatment with MQ, which is known to block endocytosis and thus uptake of hemoglobin (78), as well as CQ-mediated Hb accumulation (46), prevented both effects. We therefore believe that the sensor degradation observed is, likely indirectly, heme-mediated, by mechanisms, which will require further investigation. Although we did not carry out western blot on parasites treated with AQ or PYRO, given the similar distinct ATeam ratio depletion, we believe the latter is highly likely to cause similar sensor degradation.

### Conclusion and future outlook

Here, we have established ATeam and sfpHluorin measurements in *P. falciparum* and demonstrated their feasibility to dynamically monitor changes in ATP and pH levels in either a plate reader or single-cell microscopy format. We show that, despite some limitations, ATeams and sfpHluorin are valuable tools for studying ATP and pH metabolism in *P. falciparum*. Using these new tools, we uncover distinct response patterns toward different compound classes, adding new momentum for mode of action studies. We believe that this system shows great potential to support mode of action studies of current and future anti-malaria compounds, and thus accelerate drug development, and these tools are available on request to the malaria community. Although not herein demonstrated, we wish to note that these sensors, by virtue of their genetically encoded nature, can easily be targeted to specific subcellular compartments, and thus can be used to elucidate compartment-specific effects that may have otherwise been overlooked using conventional methods.

## MATERIALS AND METHODS

### Cloning of expression and integration plasmids

For cloning, ATeam1.03-nD/nA, ATeam1.03YEMK, and sfpHluorin template sequences were amplified via forward primer conferring BamHI and AvrII, as well as reverse primer conferring XhoI and HindIII restriction sites. In the case of ATeam sequences, the primer had multiple binding sites within the template sequence leading to multiple amplification products. Amplification products were separated and extracted from agarose gel electrophoresis for further cloning of the desired product. The sequences were cloned into the pQE-30 vector via BamHI and HindIII, while cloning into the pDC2-CAM-[X]-BSD-*attP* vector was facilitated via the AvrII and XhoI restriction sites.

### Expression and purification of recombinant ATeam and sfpHluorin protein

*E. coli* strain M15 carrying pQE-30[ATeam1.03-nD/nA] or pQE-30[ATeam1.03YEMK] were grown in lysogeny broth (LB), as described in reference (79) (1 g/L NaCl, 5 g/L yeast extract, and 1 g/L tryptone), at 37°C overnight to inoculate 1 L LB to an OD_600_ of 0.2 with subsequent incubation to OD_600_ of 0.6 at 37°C. Protein expression was induced via isopropyl β-D-1-thiogalactopyranoside to a final concentration of 0.2 mM overnight at 24°C. Cells were collected via centrifugation at 11,448 *g* for 15 minutes at 4°C and stored at −20°C until purification. Cells were thawed in lysis buffer (100 mM Tris-HCl, 200 mM NaCl, and 10 mM imidazole, pH 8.0) enriched with 150 nM pepstatin, 4 nM cystatin, and 100 µM phenylmethylsulfonylfluoride. Cells were lysed using lysozyme and DNaseI for 30 minutes on ice, disrupted by sonication, and collected via centrifugation at 38,000 *g* for 30 minutes. The supernatant was loaded onto a nickel nitrilotriacetic acid (Ni-NTA) column equilibrated in lysis buffer for affinity chromatography. After washing, proteins were eluted with increasing concentrations of imidazole (20–500 mM). Fractions containing predominantly ATeam protein were pooled and applied to a HiLoad 16/600 Superdex 200 column (GE Healthcare) equilibrated with 20 mM Tris-HCl and 150 mM NaCl, pH 8.0 for size-exclusion chromatography. Fractions containing ATeam protein were pooled and concentrated in a 30 kDa cutoff Vivaspin tube, supplemented with 20% (vol/vol) glycerol, and stored at −80°C in single-use aliquots. sfpHluorin in pQE-30 was expressed and purified in *E. coli* M15 in the same way as ATeam protein with the following exceptions: protein expression was induced at 37°C; lysis was conducted in HEPES buffer (50 mM HEPES and 300 mM NaCl, pH 7.5); elution from Ni-NTA was conducted with 10–500 mM imidazole; size-exclusion chromatography was conducted in HEPES buffer; protein was concentrated using 10 kDa cutoff Vivaspin tubes; protein was stored at −20°C in HEPES buffer. The concentration of proteins was either determined according to reference (80) or using the mVenus extinction coefficient as described by Imamura et al. (22).

### Plate reader measurements of purified ATeam protein

Purified ATeam protein was mixed to a final concentration of 1 µM in ATeam buffer (100 mM HEPES, 50 mM KCl, and 0.5 mM MgCl_2_, pH 7.34), except for pH response analyses, in which buffers contained 50 mM KCl, 0.5 mM MgCl_2_, and 100 mM of either MES (pH 5–6.5), HEPES (pH 7–8), or CHES (pH 8.5–9). Measurements were performed on a Clariostar plate reader (BMG Labtech, Ortenberg, Germany) preheated to 37°C. Emission ratios were calculated after background subtraction of emission at 527/10 nm and emission at 475/10 nm after excitation at 435/10 nm, respectively. Dynamic measurements over time were collected every 10 s, accordingly. Spectral scans were collected from 463 to 600 nm for each nanometer after excitation at 435/10 nm. Mean values of *n* = 3 experiments with independently produced proteins are shown.

### Plate reader measurements of purified sfpHluorin protein

Purified sfpHluorin protein was equilibrated in buffers to a final concentration of 1 µM with pH varying from 5.0 to 9.0 (pH 5.0–6.5: 10 mM MES-KOH, 100 mM NaCl, and 5 mM EDTA; pH 7.0–8.0: 100 mM HEPES-KOH, 100 mM NaCl, and 5 mM EDTA; pH 8.5–9.0: 100 mM Tris-HCl, 100 mM NaCl, and 5 mM EDTA) at 37°C before use. Measurements were performed on a Clariostar plate reader (BMG Labtech, Ortenberg, Germany) preheated to 37°C. Excitation scan was recorded in the range from 340 to 490 nm with emission sensing at 530/40 nm. Mean values of *n* = 3 experiments with independently produced proteins are shown.

### Drug-sensor interaction studies

Purified sensor proteins were equilibrated in ATeam (100 mM HEPES, 50 mM KCl, and 0.5 mM MgCl_2_, pH 7.34) or sfpHlourin (100 mM potassium phosphate, 100 mM NaCl, and 0.5 mM Na_2_-EDTA, pH 7.0) buffer at 37°C to a final concentration of 1 µM together with 10× stock solutions ranging from 100 to 10 µM of compound, before use. Measurements were performed on a Clariostar plate reader (BMG Labtech, Ortenberg, Germany). ATeam emission ratio was calculated after background subtraction of emission at 527/10 nm and emission at 475/10 nm after excitation at 435/10 nm, respectively. sfpHluorin excitation ratio was calculated from 390/15 and 482/16 nm excitation and 530/20 nm emission, respectively. Mean values of *n* = 3 experiments with independently produced proteins are shown.

### Stable integration of sensors into NF54*attB*

We used the NF54*attB* strain lacking a selectable marker (39) for *attB-attP* recombination of *attP* plasmids, containing the sensor sequences, within the cg6 gene using the mycobacteriophage Bxb1 integrase as described elsewhere (40). Briefly, 200 µL of 5% iRBCs were transfected with 50 µg pDC2*attP* plasmids, including blasticidin S deaminase selectable marker, conferring resistance to blasticidin S, calmodulin promotor, and the respective sensor sequence, in addition to 50 µg of the integrase-encoding pINT plasmid, including neomycin selectable marker, conferring resistance to Geneticin (G418). For transfection, cells were mixed with plasmids and cytomix (120 mM KCl, 0.15 mM CaCl_2_, 2 mM EGTA, 5 mM MgCl_2_, 10 mM K_2_HPO_4_/KH_2_PO_4_, and 25 mM HEPES, pH 7.6), transferred to a 2 mm electroporation cuvette and electroporated (310 V, 950 µF, and capacitance ∞). Transfectants were selected with 2.5 µg/mL blasticidin (until clonal selection) and 125 µg/mL G418 (5 days). Clonal parasite lines were generated using limiting dilution. Stable integration was verified using PCR as described by Schuh et al. (30), binding inside the genomic cg6 and the integrated bsd gene.

### *P. falciparum* cell culture

Parasites were cultured as described elsewhere (81). In brief, parasites were propagated in A^+^ red blood cells in complete media (CM) composed of RPMI 1640 medium supplemented with 0.5% Albumax, 9 mM glucose, 0.2 mM hypoxanthine, 2.1 mM L-glutamine, 25 mM HEPES, and 22 µg/mL gentamicin at 3.3% hematocrit and 37°C under 3% O_2_ and 3% CO_2_. Cells were synchronized via 5% sorbitol at least three times before each measurement. Cells were treated with sorbitol in their developmental cycles preceding the cycle of the measurement.

### *In vitro P. falciparum* drug susceptibility assay

Half maximal effective concentration (EC_50_) of PYRO against *P. falciparum* NF54*attB* cell line was performed using SYBR Green I-based fluorescence assays according to Ekland et al. (82). Serial double dilutions of the compound in CM were performed in 96-well plates (black, half area, μClear, Greiner Bio-One GmbH, Frickenhausen, Germany). Synchronized ring-stage parasites were added to each well to 0.15% parasitemia and 1.25% hematocrit and incubated at 37°C for 48 h. Subsequently, SYBR Green in lysis buffer (20 mM Tris-HCl, 5 mM EDTA, 0.16%, wt/vol saponin, and 1.6% vol/vol Triton X-100) was added to each well for 24 h at RT in the dark. Fluorescence was measured in a Clariostar plate reader (BMG Labtech, Ortenberg, Germany) at 494 nm excitation and 530 nm emission sensing. Curve fitting of the growth inhibition against log compound concentration with a variable slope sigmoidal function led to the EC_50_ value.

### Trophozoite enrichment via MACS

Briefly, sorbitol-synchronized iRBCs were transferred to MACS cell separation LD columns (Miltenyi Biotec, Bergisch Gladbach, Germany), washed, and eluted in CM. For measurements using live-cell microscopy, cells were washed and resuspended in Ringer’s solution (122.5 mM NaCl, 5.4 mM KCl, 1.2 mM CaCl_2_, 0.8 mM MgCl_2_, 11 mM D-glucose, 25 mM HEPES, and 1 mM NaH_2_PO_4_, pH 7.4) to a density leading to approximately 30 parasites per field of view. For plate reader measurements, cells were counted using the improved Neubauer hemocytometer (Brand GmbH, Wertheim, Germany) and diluted to the desired parasite count.

### Western blot analysis

Western blot analysis was conducted with MACS-enriched trophozoite-iRBCs. For each sample, 5 million cells were either first incubated with 100× compound stock solution or vehicle control in CM or directly washed with PBS and cOmplete Protease Inhibitor Cocktail Tablets (Roche Diagnostics GmbH, Mannheim, Germany) and lysed using M-PER buffer (ThermoFisher Scientific, Waltham, MA, USA). Cell debris was pelleted, and the supernatant was incubated at 95°C with 4× sample buffer + DTT and separated using sodium dodecyl sulfate-polyacrylamide gel electrophoresis according to reference (83). Proteins were blotted on polyvinylidene difluoride membrane. The membrane was stained with Ponceau S protein stain, washed, blocked in 5% milk, and probed with α-GFP (1:1,000 in 5% milk; Roche Diagnostics GmbH, Mannheim, Germany) for GFP-based sensor probing, followed by secondary α-mouse IgG antibodies (1:10,000 in 5% milk; Dianova, Hamburg, Germany). Equal loading via housekeeping genes was verified via α-aldolase or α-HSP90 western blot, after GFP probing. All solutions for western blot staining were dissolved in Tris-buffered saline with 0.05% polysorbate 20.

### Measurements of sensor cell lines via plate reader

For plate reader measurements, 2 million NF54*attB*^[ATeam1.03YEMK]^/NF54*attB*^[ATeam1.03-nD/nA]^ or 1 million NF54*attB*^[sfpHluorin]^ MACS-enriched trophozoite-iRBCs were washed in Ringer’s solution, mixed in 384-well small volume plates (Greiner Bio-One GmbH, Frickenhausen, Germany), and measured in Clariostar plate reader (BMG Labtech, Ortenberg, Germany) at 37°C. For NF54*attB*^[ATeam1.03YEMK]^ and NF54*attB*^[ATeam1.03-nD/nA]^, measurement settings were chosen as described for recombinant ATeam protein earlier. For NF54*attB*^[sfpHluorin]^, spectral excitation scan was collected via emission sensing at 530/40 nm and excitation from 350 to 490 nm for every nanometer with 10 nm bandwidth. The ratio was calculated after background subtraction of emission at 530/20 nm and excitation at 390/15 or 482/16 nm, respectively. Dynamic measurements over time were monitored every 10 s.

### Measurements of sensor cell lines via live-cell microscopy

For imaging, Axio Observer.Z1/7 microscope (Zeiss, Oberkochen, Germany) with plan-apochromat 63×/1.40 oil immersion DIC M27 objective and Axiocam 506 camera were used. For measurements, CM was washed off with Ringer’s solution, and parasites were allowed to settle on pre-heated ibidiTreat µ-Dish 35 mm Quad microcopy dishes (ibidi GmbH, Gräfelfing, Germany) at 37°C. The emission ratio of NF54*attB*^[ATeam1.03YEMK]^ was measured using Zeiss filter set 47 (EX BP 436/20, BS FT 455, EM BP 480/40) and 48 (EX BP 436/20, BS FT 455, EM BP 535/30) for CFP and CFP-YFP-FRET signal, respectively. Colibri 7 LED module 430 nm was used for excitation. FRET ratio equals background-corrected YFP signal at 535 nm divided by background-corrected CFP signal at 480 nm. The excitation ratio of NF54*attB*^[sfpHluorin]^ was measured using Zeiss filter set 38 HE without excitation filter [BS FT 495 (HE), EM BP 525/50 (HE)]. The excitation ratio equals background-corrected GFP signal at 525 nm and excitation at 385 nm divided by background-corrected GFP signal at 525 nm and excitation at 475 nm. ROIs were defined using a semi-automatic ImageJ script. The background area was selected manually. Image analysis was conducted via ImageJ Fiji (84) 1.54f.

### Statistical analysis

Statistical analyses were performed via GraphPad Prism 10.02. Analysis of drug effects on sensor-expressing cell lines was conducted via repeated measure two-way ANOVA using a general linear model for the analysis of the selected randomized block design. Correction for multiple comparisons was conducted using the false discovery rate with the two-staged step-up method described by Benjamini et al. (44). The desired FDR was set at 5%. Calibration curves for interpolation of pH values were generated using a sigmoidal, four-parameter logistic model.

## SUPPLEMENTAL MATERIAL

Supplemental material aac.01690-23-s0001.docx Figures S1 to S11; Tables S1 to S10.

ASM does not own the copyrights to Supplemental Material that may be linked to, or accessed through, an article. The authors have granted ASM a non-exclusive, world-wide license to publish the Supplemental Material files. Please contact the corresponding author directly for reuse.
