# Supplementary material for: Real-time measurements of ATP dynamics via ATeams in Plasmodium falciparum reveal drug-class-specific response patterns
Source: Antimicrob Agents Chemother. 2024 Mar 19;68(5):e01690-23. doi: 10.1128/aac.01690-23 (PMC11064498; doi:10.1128/aac.01690-23)
Supplement: Supplemental material — Figures S1 to S11; Tables S1 to S10. [file aac.01690-23-s0001.docx]

**SUPPLEMENTAL MATERIALS**


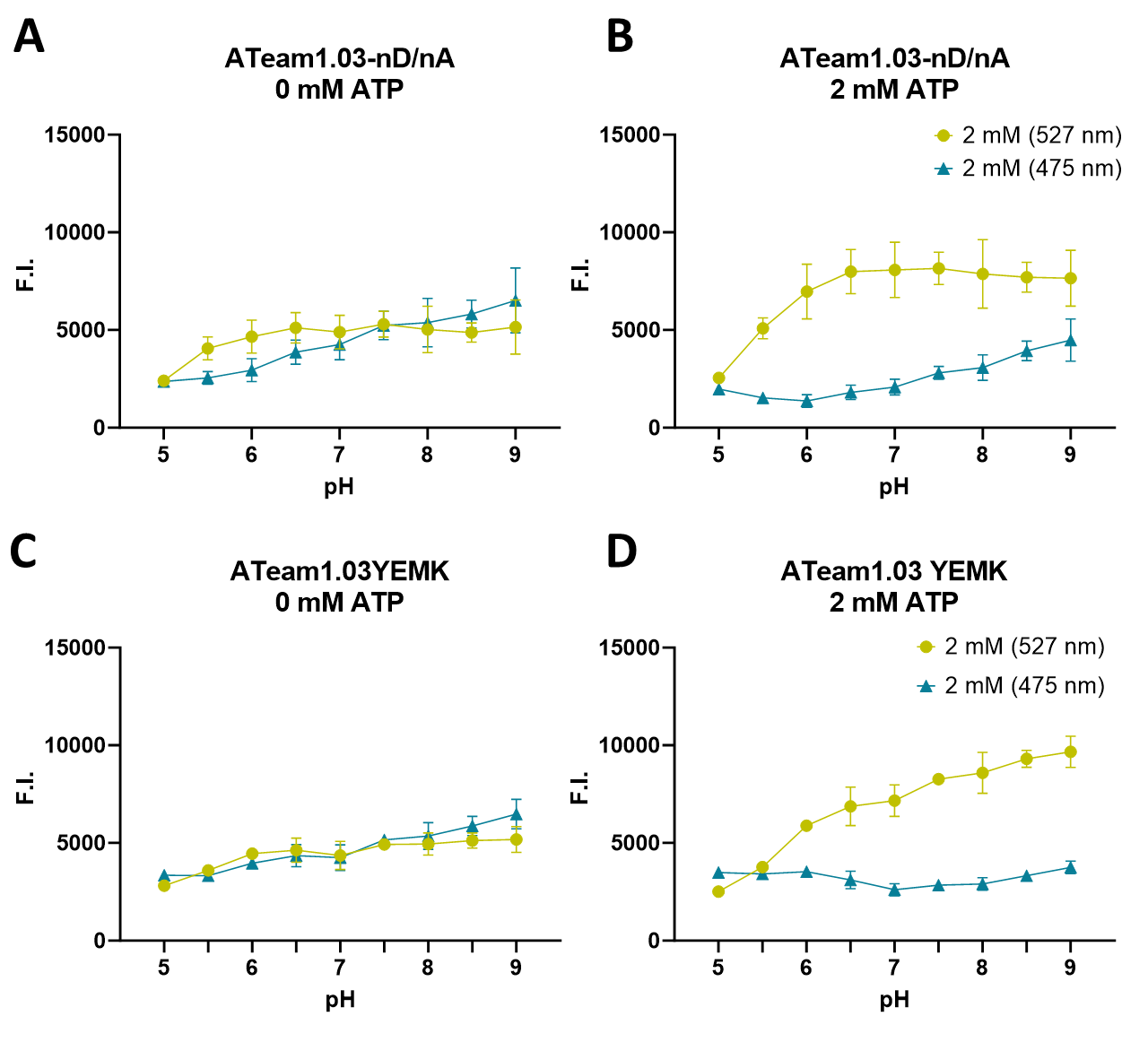


**Fig S1: ATeam1.03-nD/nA shows less pH stability compared to ATeam1.03YEMK.** A: CFP emission of ATeam1.03-nD/nA is effected by pH in the physiological range from pH 6 to 8; B: Increased dynamic range of ATeam1.03-nD/nA in response to 2 mM ATP is accompanied with decreased CFP emission; C: CFP and YFP fluorescence of ATeam1.03YEMK in the absence of ATP is stable in the physiological relevant range of pH 6 to 8; D: Ratio [527/475] decrease of ATeam1.03YEMK from pH 7 to 5 is accompanied with decreasing and increasing YFP and CFP fluorescence, respectively; Measurements were conducted via excitation at 435 nm and emission sensing at 527 nm for YFP and 475 nm for CFP. ATP was solved in equimolar solution of MgCl_2_. 1 µM recombinant protein was used. Mean values of n = 3 independent experiments are shown. Error bars indicate SD.


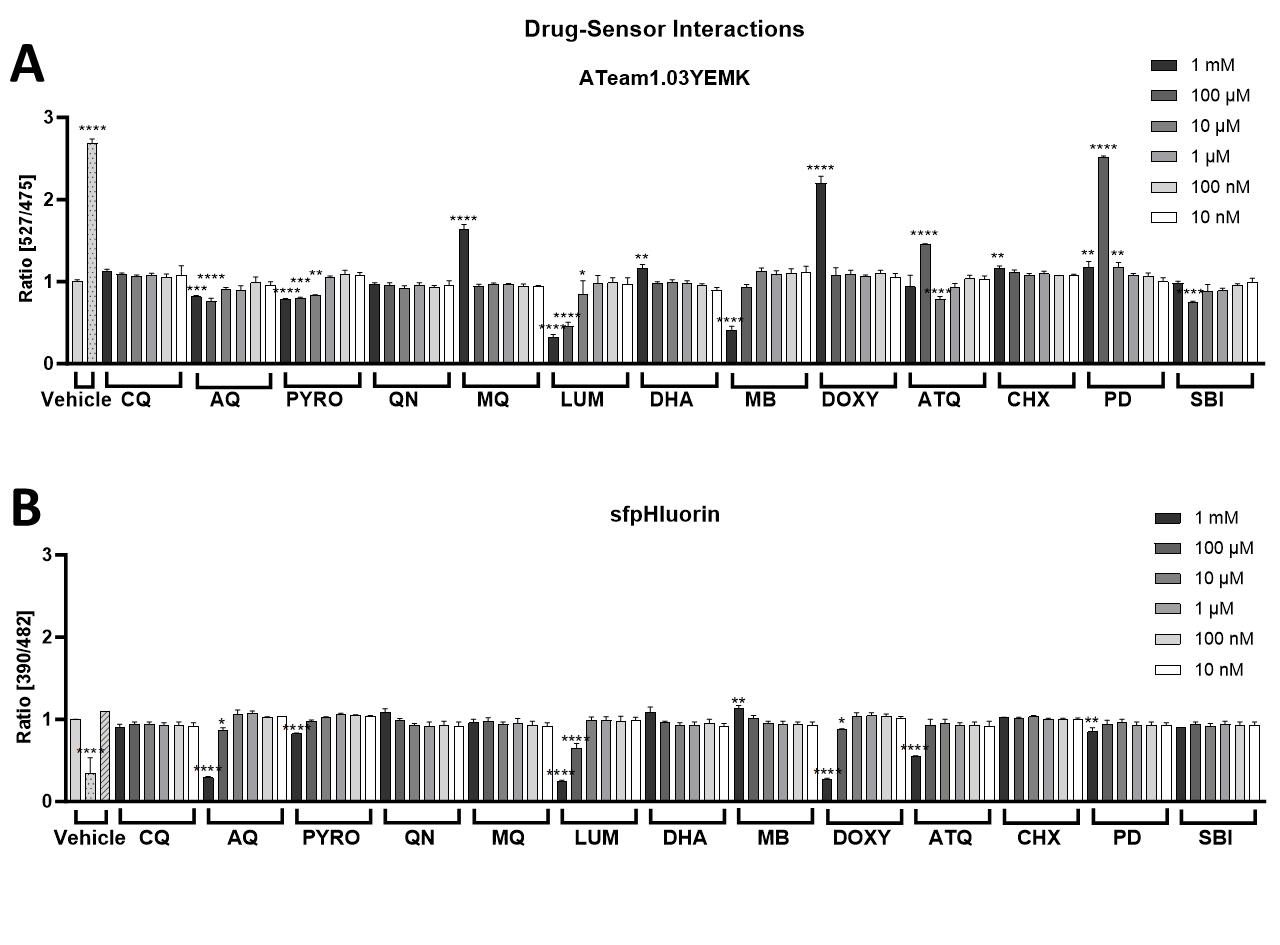


**Fig. S2: ATeam1.03YEMK and sfpHluorin drug-sensor interactions.** Recombinant sensor protein was incubated with the indicated drugs (1 mM, 100 µM, 10 µM, 1 µM, 100 nM, 10 nM); A: ATeam1.03YEMK measurements included vehicle treatment with 10 mM ATP dissolved in equimolar MgCl_2_ solution (dotted); B: sfpHluorin measurements included vehicle treatment with buffers set to pH 5 (dotted) and pH 9 (diagonal); Chloroquine (CQ), amodiaquine (AQ), pyronaridine (PYRO), quinine (QN), mefloquine (MQ), lumefantrine (LUM), dihydroartemisinin (DHA), methylene blue (MB), doxycycline (DOXY), atovaquone (ATQ), cycloheximide (CHX), plasmodione (PD), SBI 0797750 (SBI). Background-corrected emission (A) or excitation (B) ratio relative to control is shown. Sensors were incubated for 5 min. Mean ratio of n = 3 independent experiments is shown. Error bars indicate SD. Ordinary one-way ANOVA with Dunnett’s multiple comparisons testing was used. *: p < 0.05; **: p < 0.01; ***: p < 0.001; ****: p < 0.0001.


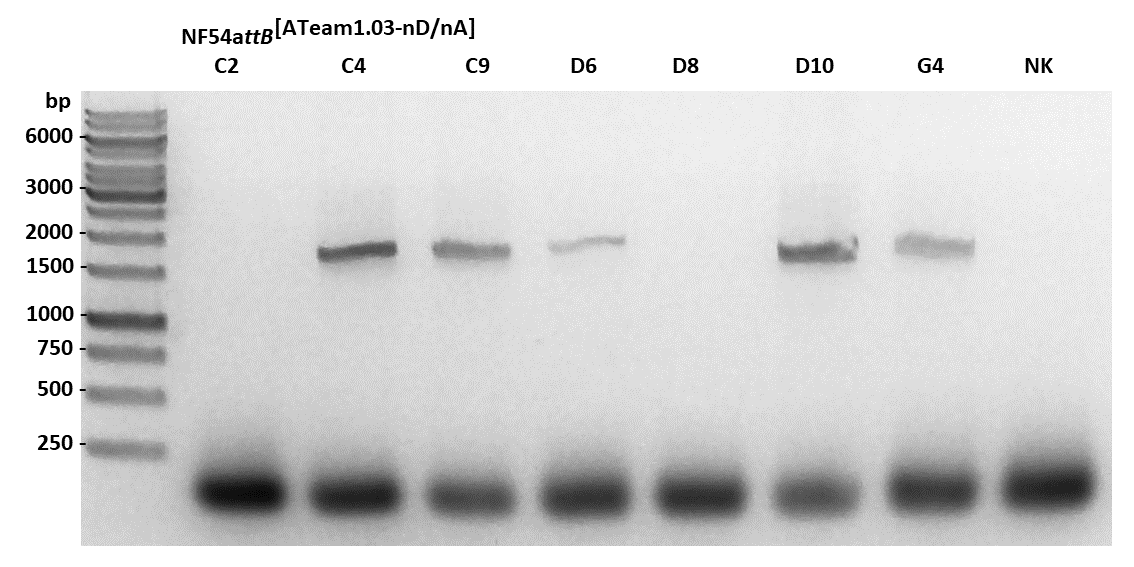


**Fig. S3: Stable integration of ATeam1.03-nD/nA in NF54*attB*.** Representative clonal cell-lines show PCR product based on genomic cg6 and integrated BSD genes, and thereby, demonstrating successful sensor integration.


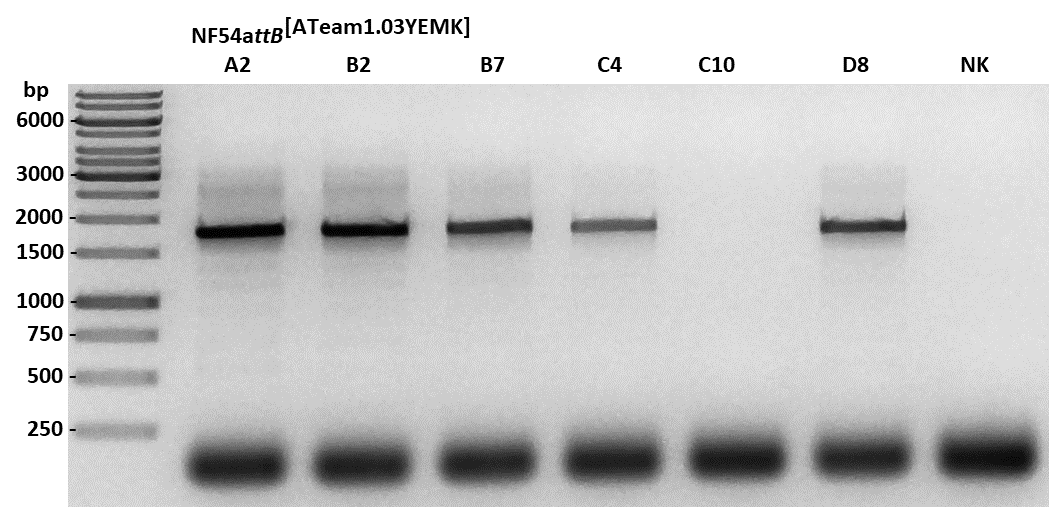


**Fig. S4: Stable integration of ATeam1.03YEMK in NF54*attB*.** Representative clonal cell-lines show PCR product based on genomic cg6 and integrated BSD genes, and thereby, demonstrating successful sensor integration.


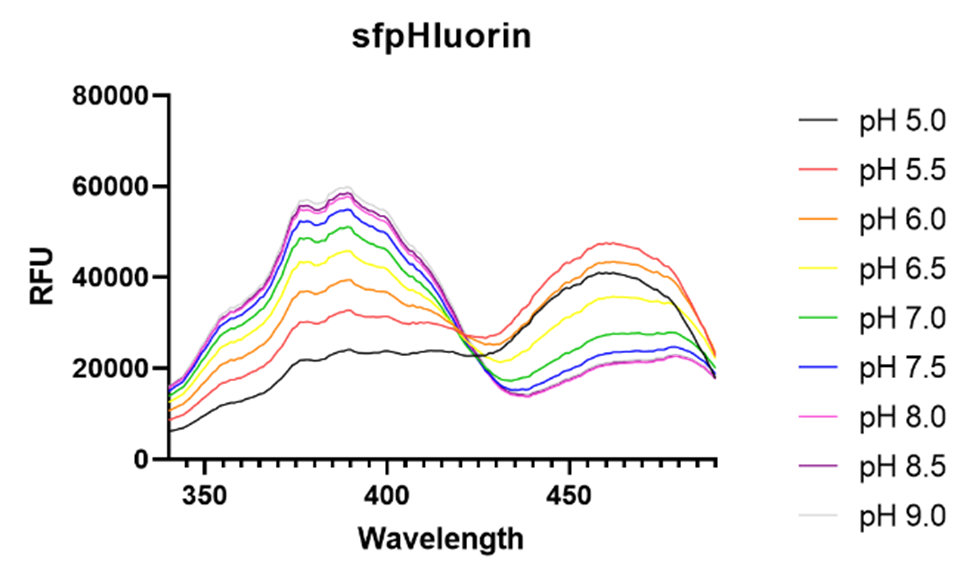


**Fig. S5: NF54*attB*^[sfpHluorin]^ *in vitro* calibration in a plate reader format.** Excitation spectra from 350 to 490 nm with emission sensing at 530 nm show pH responsive peaks around 390 and 482 nm. Error bars were omitted for clarity. Means of n = 3 independent experiments are shown.

**
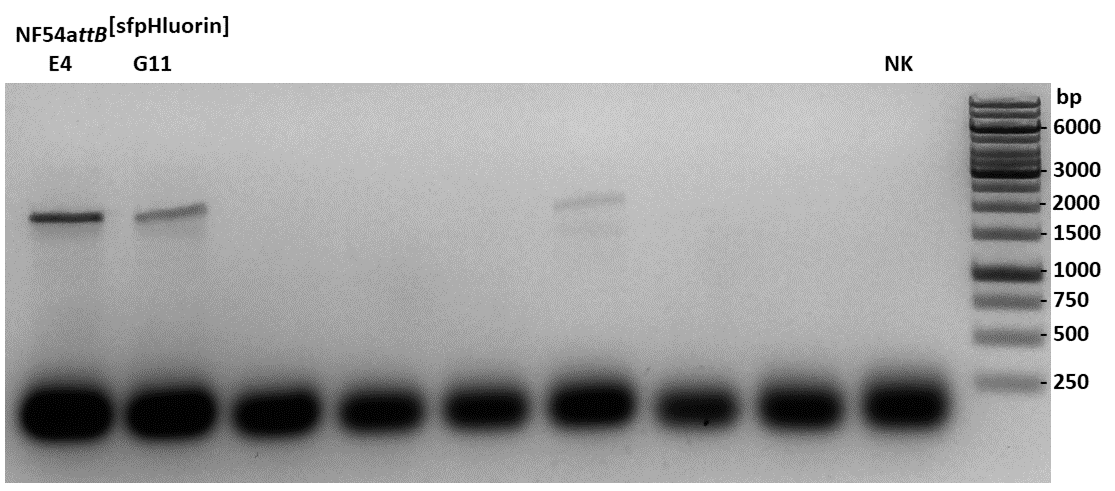
**

**Fig. S6: Stable integration of sfpHluorin in NF54*attB*.** Representative clonal cell-lines show PCR product based on genomic cg6 and integrated BSD genes, and thereby, demonstrating successful sensor integration.


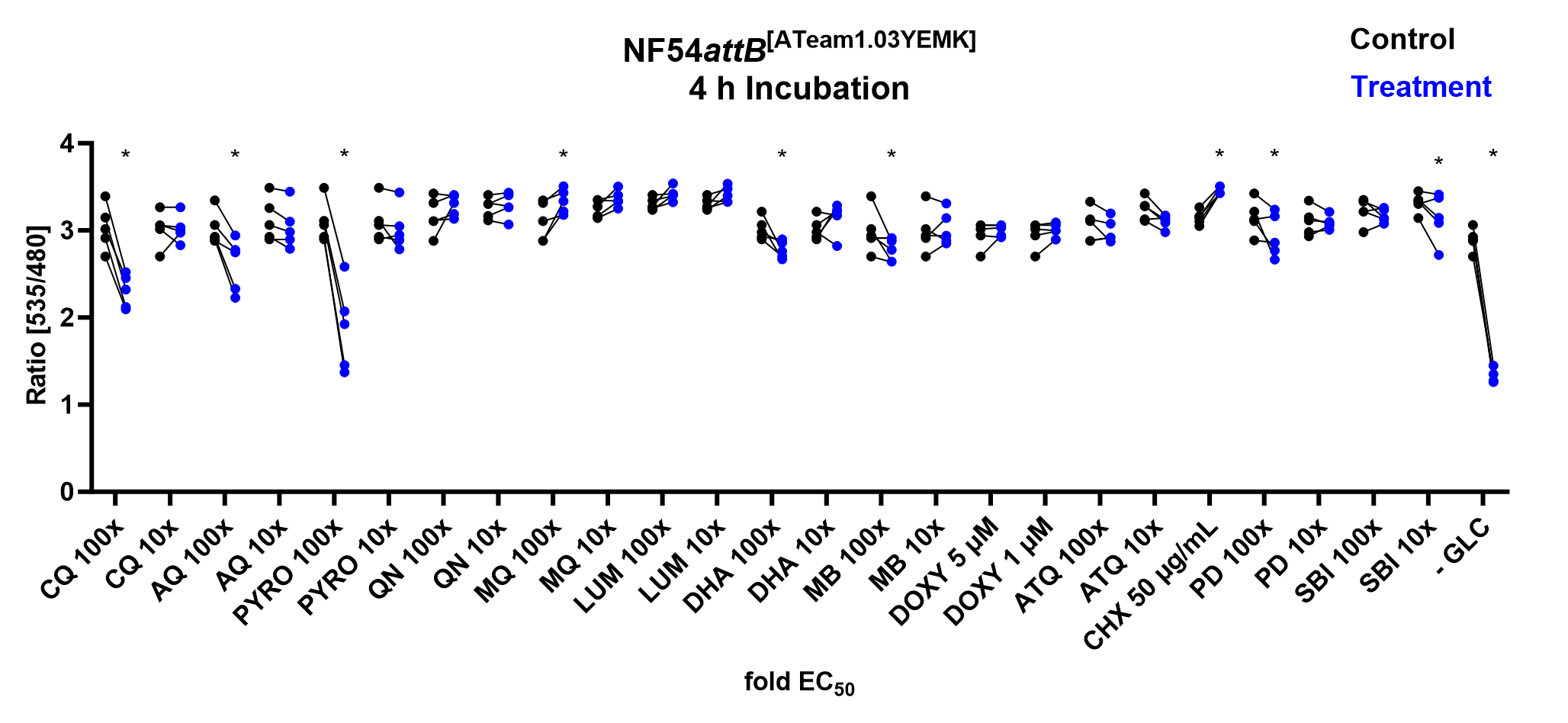


**Fig. S7: NF54*attB*^[ATeam1.03YEMK]^ shows distinct ratio changes in response towards different drug classes after 4 h incubation.** Fluorescence microscopic measurement of MACS-enriched trophozite infected red blood cells. Each measurement corresponds to the 535 nm to 480 nm emission ratio after excitation at 430 nm. Mean of 100 single cell analyses of compound intervention (Treatment) and contemporaneous vehicle control (Control) with n = 5 independent experiments are shown. Treatment groups included chloroquine (CQ), amodiaquine (AQ), pyronaridine (PYRO), quinine (QN), mefloquine (MQ), lumefantrine (LUM), dihydroartemisinin (DHA), methylene blue (MB), doxycycline (DOXY), atovaquone (ATQ), cycloheximide (CHX), plasmodione (PD), SBI 0797750 (SBI), and glucose starvation (-GLC). Cells were starved from GLC through washing in PBS just before measurement. If not indicated otherwise, compounds were applied with 100x and 10x EC_50_ concentration. * indicates discovery with FDR = 5 %.


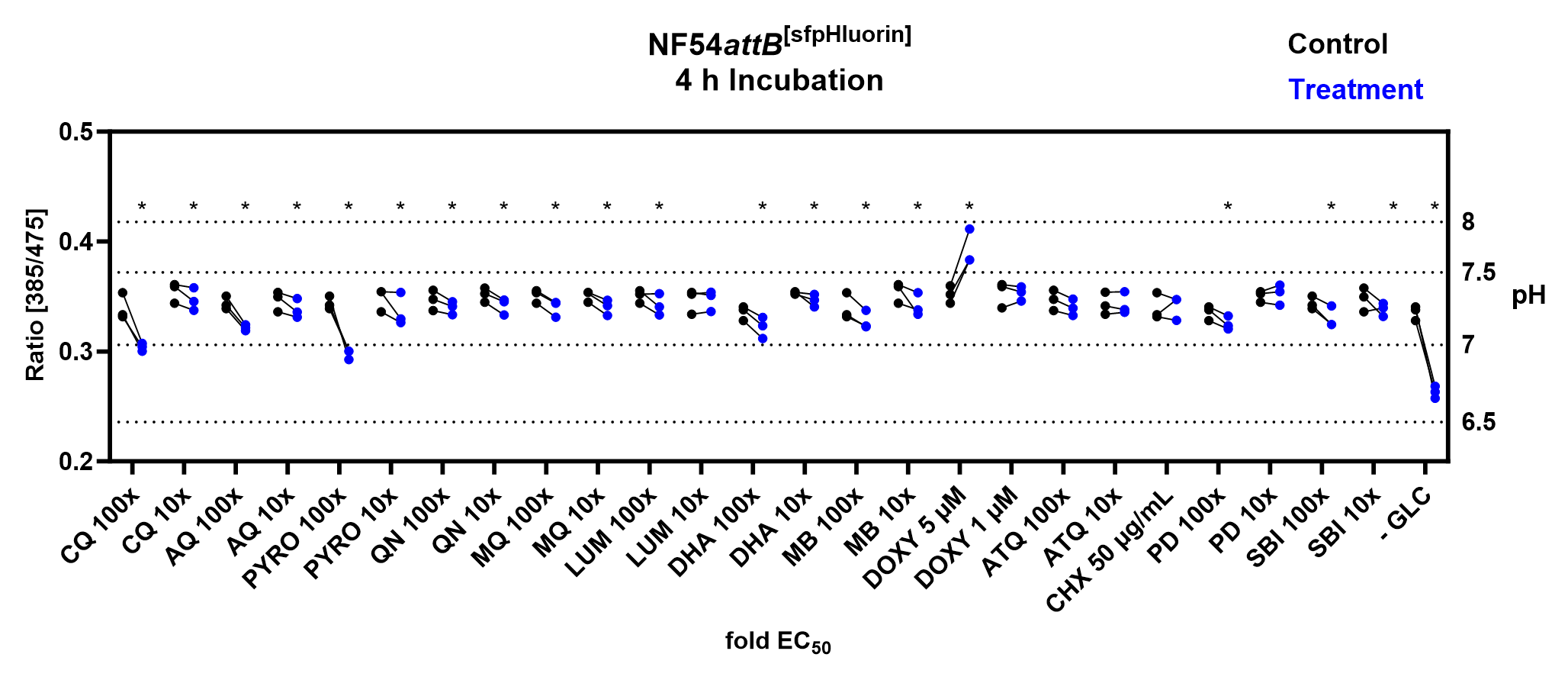


**Fig. S8:** **NF54*attB*^[sfpHluorin]^ shows predominantly marginal ratio changes in response towards different drug classes after 4 h incubation time.** Fluorescence microscopic measurement of MACS-enriched trophozite infected red blood cells. Each measurement corresponds to the 385 to 475 nm excitation ratio at 525 nm emission. Mean of 100 single cell analyses of compound intervention (Treatment) and concurrent vehicle control (Control) with n = 3 independent experiments are shown. Treatment groups included chloroquine (CQ), amodiaquine (AQ), pyronaridine (PYRO), quinine (QN), mefloquine (MQ), lumefantrine (LUM), dihydroartemisinin (DHA), methylene blue (MB), doxycycline (DOXY), atovaquone (ATQ), cycloheximide (CHX), plasmodione (PD), SBI 0797750 (SBI), and glucose starvation (-GLC). Cells were starved from GLC through washing in PBS just before measurement. If not indicated otherwise, compounds were applied with 100x and 10x EC_50_ concentration. * indicates discovery with FDR = 5 %.


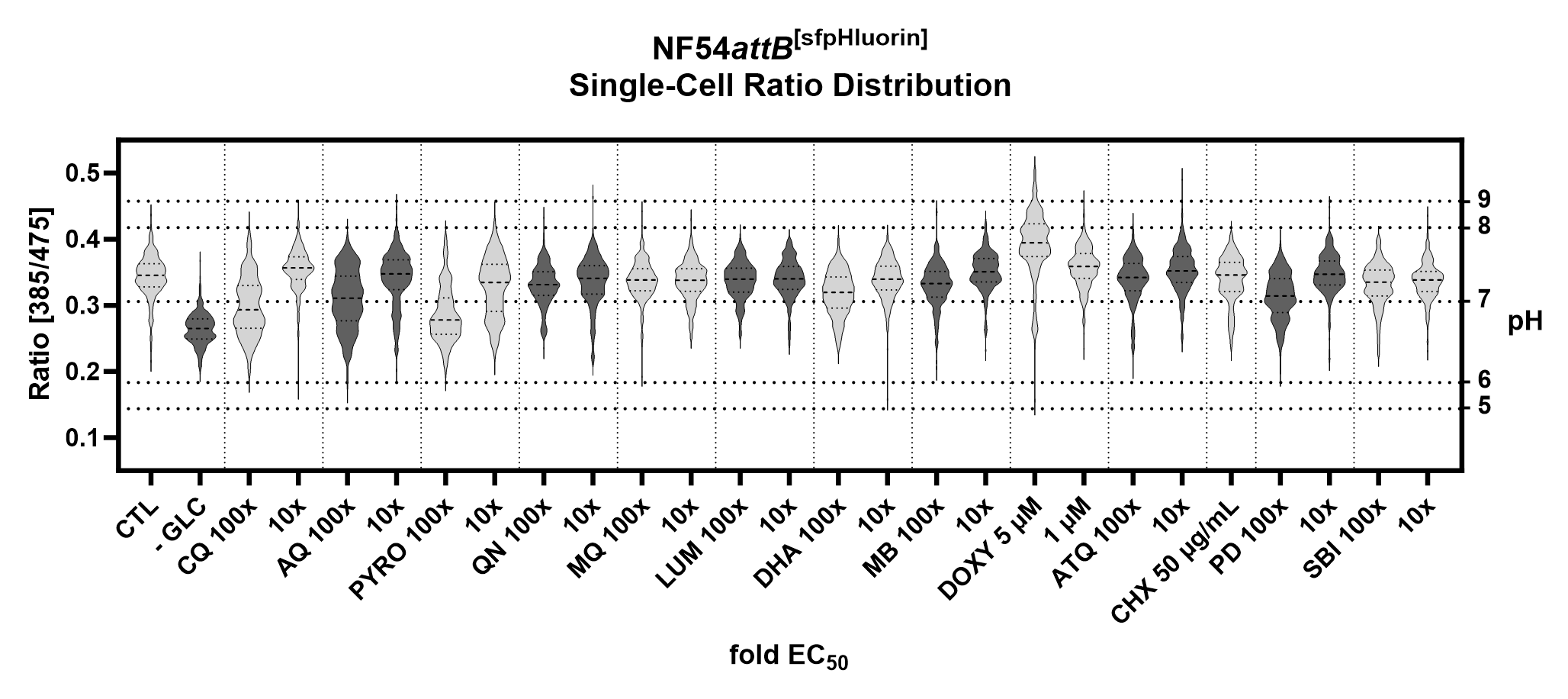


**Fig. S9: NF54*attB*^[sfpHluorin]^ shows distinct single-cell excitation ratio distribution in response towards different drug classes.** Fluorescence microscopic measurement of MACS-enriched trophozite-iRBCs. Each measurement corresponds to the 385 to 475 nm excitation ratio at 525 nm emission. Pool of n = 3 independent experiments with each 100 single cell analyses is shown. Treatment groups included chloroquine (CQ), amodiaquine (AQ), pyronaridine (PYRO), quinine (QN), mefloquine (MQ), lumefantrine (LUM), dihydroartemisinin (DHA), methylene blue (MB), doxycycline (DOXY), atovaquone (ATQ), cycloheximide (CHX), plasmodione (PD), SBI 0797750 (SBI), and glucose starvation (-GLC). Cells were starved from GLC through washing in PBS just before measurement. If not indicated otherwise, compounds were applied with 100x and 10x EC_50_ concentration.


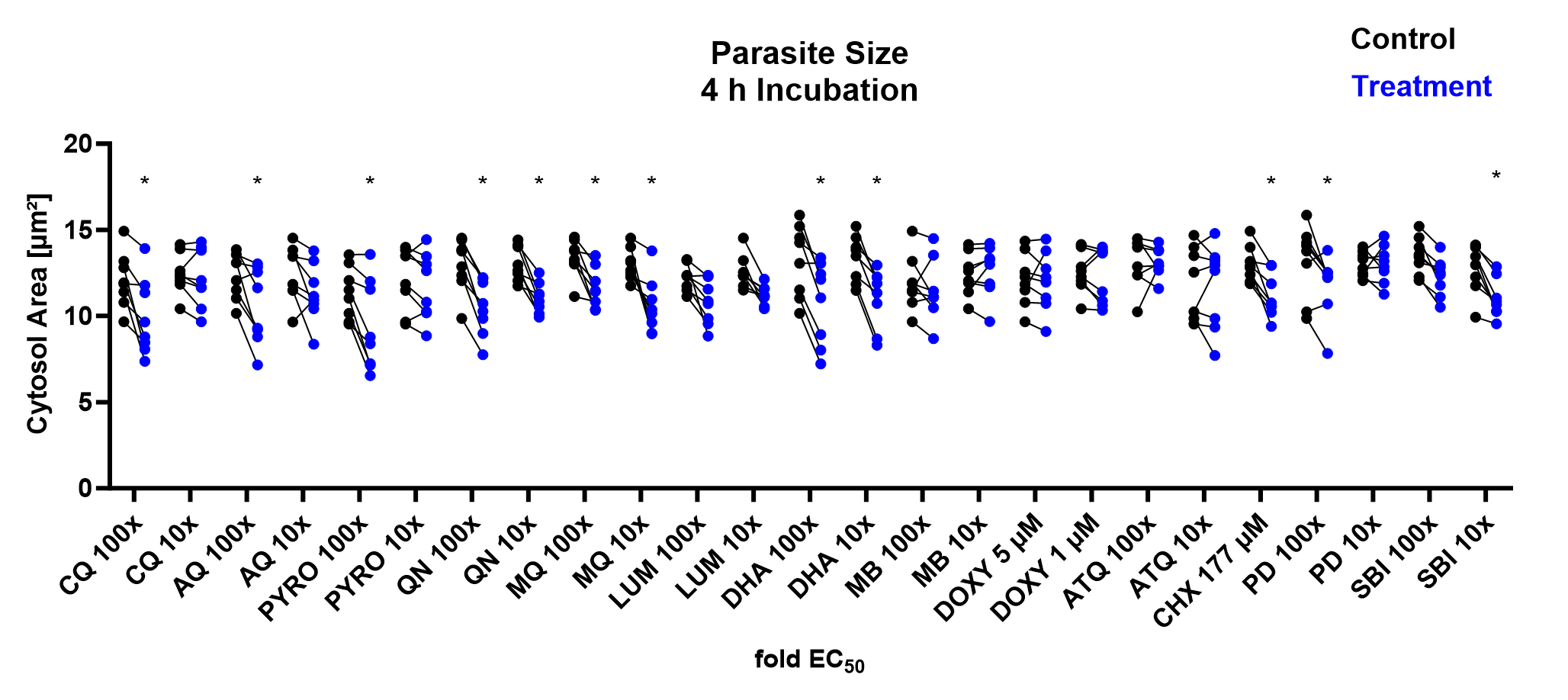


**Fig. S10 NF54*attB*^[ATeam1.03YEMK]^ and NF54*attB*^[sfpHluorin]^ show decreased parasite size in response towards different drug classes after 4 h incubation.** The mean cytosol size of 100 MACS-enriched trophozite infected red blood cells derived from automated ROI definition from fluorescence microscopic measurements of NF54*attB*^[ATeam1.03YEMK]^ (n = 5) and NF54*attB*^[sfpHluorin]^ (n = 3) is combined. Analyses of compound intervention (Treatment) and concurrent vehicle control (Control) with in total n = 8 independent experiments are shown. Treatment groups included chloroquine (CQ), amodiaquine (AQ), pyronaridine (PYRO), quinine (QN), mefloquine (MQ), lumefantrine (LUM), dihydroartemisinin (DHA), methylene blue (MB), doxycycline (DOXY), atovaquone (ATQ), cycloheximide (CHX), plasmodione (PD), and SBI 0797750 (SBI). If not indicated otherwise, compounds were applied with 100x and 10x EC_50_ concentration. * indicates discovery with FDR = 5 %.


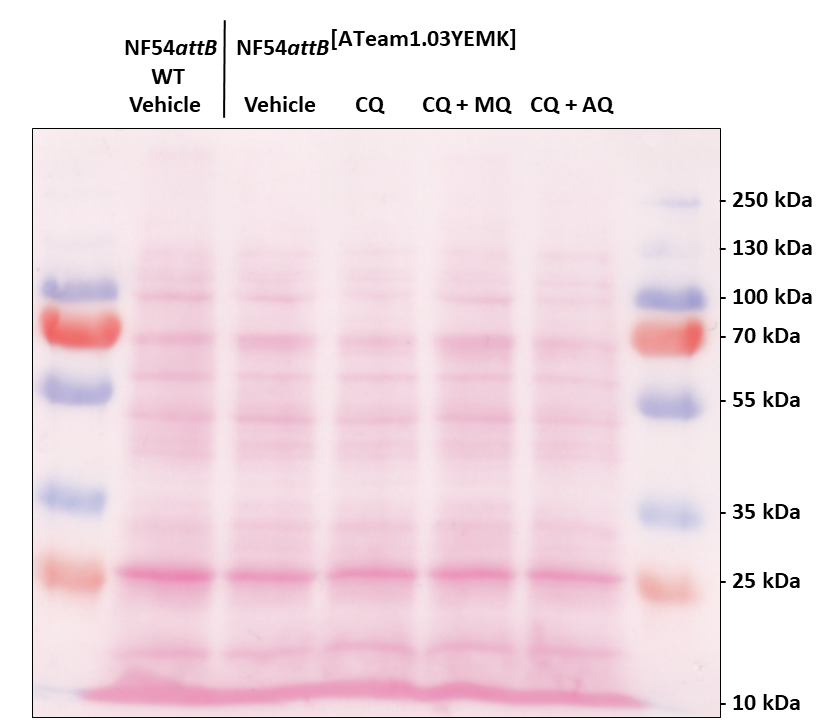


**Fig. S11: Ponceau S protein staining demonstrates sample loading.**

**Table S1: EC_50_ concentrations used for drug incubation of sensor cell lines.** EC_50_ values were either measured (PYRO) or orientated to results in the literature.

| **Compound** | **EC_50_** |
| --- | --- |
| CQ | 6.9 nM (1) |
| AQ | 3.4 nM (1) |
| PYRO | 2.84 ± 0.77 (this study) |
| QN | 66.2 nM (1) |
| MQ | 23.4 nM (1) |
| LUM | 10.7 nM (1) |
| DHA | 5 nM (2) |
| MB | 3.3 nM (3) |
| ATQ | 0.5 nM (1) |
| PD | 50 nM (4) |
| SBI | 83.8 nM (5) |

**Table S2: Two-Way ANOVA analysis of for concurrent control and treatment of NF54*attB*^[ATeam1.03YEMK]^ after 6 h incubation time with two-stage linear step-up.**

| **Treatment** | **Mean Diff.** | **Discovery?** | **q value** | **Individual P Value** |
| --- | --- | --- | --- | --- |
|  |  |  |  |  |
| CQ 100x | 1.000 | Yes | <0.0001 | <0.0001 |
| CQ 10x | 0.02959 | No | 0.3796 | 0.6928 |
| AQ 100x | 0.7095 | Yes | <0.0001 | <0.0001 |
| AQ 10x | 0.1803 | Yes | 0.0170 | 0.0175 |
| PYRO 100x | 1.608 | Yes | <0.0001 | <0.0001 |
| PYRO 10x | 0.2056 | Yes | 0.0080 | 0.0070 |
| QN 100x | -0.1170 | No | 0.0843 | 0.1205 |
| QN 10x | -0.07470 | No | 0.2120 | 0.3196 |
| MQ 100x | -0.1774 | Yes | 0.0174 | 0.0194 |
| MQ 10x | -0.1963 | Yes | 0.0104 | 0.0099 |
| LUM 100x | -0.2276 | Yes | 0.0041 | 0.0029 |
| LUM 10x | -0.2153 | Yes | 0.0061 | 0.0048 |
| DHA 100x | 0.5730 | Yes | <0.0001 | <0.0001 |
| DHA 10x | -0.1431 | Yes | 0.0489 | 0.0582 |
| MB 100x | 0.3145 | Yes | <0.0001 | <0.0001 |
| MB 10x | -0.01869 | No | 0.4148 | 0.8029 |
| DOXY 5 µM | -0.01544 | No | 0.4148 | 0.8367 |
| DOXY 1 µM | -0.01360 | No | 0.4148 | 0.8559 |
| ATQ 100x | 0.1178 | No | 0.0843 | 0.1179 |
| ATQ 10x | 0.05975 | No | 0.2438 | 0.4256 |
| CHX 50 µg/mL | -0.3833 | Yes | <0.0001 | <0.0001 |
| PD 100x | 0.5180 | Yes | <0.0001 | <0.0001 |
| PD 10x | 0.07006 | No | 0.2208 | 0.3505 |
| SBI 100x | -0.06399 | No | 0.2362 | 0.3936 |
| SBI 10x | 0.1360 | No | 0.0563 | 0.0714 |
| - GLC | 1.595 | Yes | <0.0001 | <0.0001 |

**Table S3: Two-Way ANOVA analysis for concurrent control and treatment of NF54*attB*^[ATeam1.03YEMK]^ after 4 h incubation time with two-stage linear step-up.**

| **Treatment** | **Mean Diff.** | **Discovery?** | **q value** | **Individual P Value** |
| --- | --- | --- | --- | --- |
|  |  |  |  |  |
| CQ 100x | 0.7303 | Yes | <0.0001 | <0.0001 |
| CQ 10x | 0.0006286 | No | 0.6415 | 0.9928 |
| AQ 100x | 0.4177 | Yes | <0.0001 | <0.0001 |
| AQ 10x | 0.08382 | No | 0.2274 | 0.2301 |
| PYRO 100x | 1.216 | Yes | <0.0001 | <0.0001 |
| PYRO 10x | 0.07649 | No | 0.2550 | 0.2732 |
| QN 100x | -0.1189 | No | 0.1162 | 0.0899 |
| QN 10x | -0.02805 | No | 0.4810 | 0.6871 |
| MQ 100x | -0.2271 | Yes | 0.0035 | 0.0015 |
| MQ 10x | -0.1097 | No | 0.1232 | 0.1173 |
| LUM 100x | -0.1133 | No | 0.1232 | 0.1057 |
| LUM 10x | -0.1103 | No | 0.1232 | 0.1151 |
| DHA 100x | 0.2387 | Yes | 0.0024 | 0.0008 |
| DHA 10x | -0.1293 | No | 0.0916 | 0.0654 |
| MB 100x | 0.2240 | Yes | 0.0035 | 0.0017 |
| MB 10x | -0.03194 | No | 0.4722 | 0.6465 |
| DOXY 5 µM | -0.04523 | No | 0.3942 | 0.5163 |
| DOXY 1 µM | -0.05024 | No | 0.3793 | 0.4710 |
| ATQ 100x | 0.06981 | No | 0.2803 | 0.3171 |
| ATQ 10x | 0.1436 | No | 0.0627 | 0.0411 |
| CHX 50 µg/mL | -0.3122 | Yes | <0.0001 | <0.0001 |
| PD 100x | 0.2124 | Yes | 0.0053 | 0.0028 |
| PD 10x | 0.01526 | No | 0.5554 | 0.8264 |
| SBI 100x | 0.04988 | No | 0.3793 | 0.4742 |
| SBI 10x | 0.1692 | Yes | 0.0278 | 0.0165 |
| - GLC | 1.571 | Yes | <0.0001 | <0.0001 |

**Table S4: Two-Way ANOVA analysis for concurrent control and treatment of NF54*attB*^[sfpHluorin]^ after 6 h incubation time with two-stage linear step-up procedure.**

| **Treatment** | **Mean Diff.** | **Discovery?** | **q value** | **Individual P Value** |
| --- | --- | --- | --- | --- |
| CQ 100x | 0.04137 | Yes | <0.0001 | <0.0001 |
| CQ 10x | 7.927e-005 | No | 0.4383 | 0.9867 |
| AQ 100x | 0.03300 | Yes | <0.0001 | <0.0001 |
| AQ 10x | 0.01757 | Yes | 0.0007 | 0.0005 |
| PYRO 100x | 0.05615 | Yes | <0.0001 | <0.0001 |
| PYRO 10x | 0.02381 | Yes | <0.0001 | <0.0001 |
| QN 100x | 0.01447 | Yes | 0.0035 | 0.0036 |
| QN 10x | 0.01047 | Yes | 0.0231 | 0.0320 |
| MQ 100x | 0.008791 | Yes | 0.0426 | 0.0699 |
| MQ 10x | 0.01454 | Yes | 0.0035 | 0.0035 |
| LUM 100x | 0.008784 | Yes | 0.0426 | 0.0701 |
| LUM 10x | 0.01175 | Yes | 0.0137 | 0.0166 |
| DHA 100x | 0.02190 | Yes | <0.0001 | <0.0001 |
| DHA 10x | 0.01625 | Yes | 0.0014 | 0.0012 |
| MB 100x | 0.009468 | Yes | 0.0350 | 0.0515 |
| MB 10x | 0.003244 | No | 0.2613 | 0.4976 |
| DOXY 5 µM | -0.04388 | Yes | <0.0001 | <0.0001 |
| DOXY 1 µM | -0.001992 | No | 0.3398 | 0.6766 |
| ATQ 100x | 0.006393 | No | 0.1064 | 0.1842 |
| ATQ 10x | 0.001578 | No | 0.3567 | 0.7411 |
| CHX 50 µg/mL | 0.0006628 | No | 0.4110 | 0.8896 |
| PD 100x | 0.02671 | Yes | <0.0001 | <0.0001 |
| PD 10x | 0.003522 | No | 0.2540 | 0.4618 |
| SBI 100x | 0.01102 | Yes | 0.0187 | 0.0243 |
| SBI 10x | 0.01246 | Yes | 0.0102 | 0.0114 |
| - GLC | 0.07512 | Yes | <0.0001 | <0.0001 |

**Table S5: Two-Way ANOVA analysis for concurrent control and treatment of NF54*attB*^[sfpHluorin]^ after 4 h incubation time with two-stage linear step-up procedure.**

| **Treatment** | **Mean Diff.** | **Discovery?** | **q value** | **Individual P Value** |
| --- | --- | --- | --- | --- |
|  |  |  |  |  |
| CQ 100x | 0.7303 | Yes | <0.0001 | <0.0001 |
| CQ 10x | 0.0006286 | No | 0.6415 | 0.9928 |
| AQ 100x | 0.4177 | Yes | <0.0001 | <0.0001 |
| AQ 10x | 0.08382 | No | 0.2274 | 0.2301 |
| PYRO 100x | 1.216 | Yes | <0.0001 | <0.0001 |
| PYRO 10x | 0.07649 | No | 0.2550 | 0.2732 |
| QN 100x | -0.1189 | No | 0.1162 | 0.0899 |
| QN 10x | -0.02805 | No | 0.4810 | 0.6871 |
| MQ 100x | -0.2271 | Yes | 0.0035 | 0.0015 |
| MQ 10x | -0.1097 | No | 0.1232 | 0.1173 |
| LUM 100x | -0.1133 | No | 0.1232 | 0.1057 |
| LUM 10x | -0.1103 | No | 0.1232 | 0.1151 |
| DHA 100x | 0.2387 | Yes | 0.0024 | 0.0008 |
| DHA 10x | -0.1293 | No | 0.0916 | 0.0654 |
| MB 100x | 0.2240 | Yes | 0.0035 | 0.0017 |
| MB 10x | -0.03194 | No | 0.4722 | 0.6465 |
| DOXY 5 µM | -0.04523 | No | 0.3942 | 0.5163 |
| DOXY 1 µM | -0.05024 | No | 0.3793 | 0.4710 |
| ATQ 100x | 0.06981 | No | 0.2803 | 0.3171 |
| ATQ 10x | 0.1436 | No | 0.0627 | 0.0411 |
| CHX 50 µg/mL | -0.3122 | Yes | <0.0001 | <0.0001 |
| PD 100x | 0.2124 | Yes | 0.0053 | 0.0028 |
| PD 10x | 0.01526 | No | 0.5554 | 0.8264 |
| SBI 100x | 0.04988 | No | 0.3793 | 0.4742 |
| SBI 10x | 0.1692 | Yes | 0.0278 | 0.0165 |
| - GLC | 1.571 | Yes | <0.0001 | <0.0001 |

**Table S6: Two-Way ANOVA analysis of parasite size for concurrent control and treatment of NF54*attB*^[ATeam1.03YEMK]^ and NF54*attB*^[sfpHluorin]^ parasites after 6 h incubation time with two-stage linear step-up procedure.**

| **Treatment** | **Mean Diff.** | **Discovery?** | **q value** | **Individual P Value** |
| --- | --- | --- | --- | --- |
|  |  |  |  |  |
| CQ 100x | 2.145 | Yes | <0.0001 | <0.0001 |
| CQ 10x | 0.2619 | No | 0.2672 | 0.5371 |
| AQ 100x | 1.735 | Yes | <0.0001 | <0.0001 |
| AQ 10x | 1.163 | Yes | 0.0039 | 0.0067 |
| PYRO 100x | 1.924 | Yes | <0.0001 | <0.0001 |
| PYRO 10x | 0.4574 | No | 0.1566 | 0.2817 |
| QN 100x | 2.465 | Yes | <0.0001 | <0.0001 |
| QN 10x | 2.010 | Yes | <0.0001 | <0.0001 |
| MQ 100x | 1.775 | Yes | <0.0001 | <0.0001 |
| MQ 10x | 2.427 | Yes | <0.0001 | <0.0001 |
| LUM 100x | 1.475 | Yes | 0.0005 | 0.0006 |
| LUM 10x | 1.349 | Yes | 0.0012 | 0.0017 |
| DHA 100x | 2.428 | Yes | <0.0001 | <0.0001 |
| DHA 10x | 2.287 | Yes | <0.0001 | <0.0001 |
| MB 100x | 0.4181 | No | 0.1706 | 0.3249 |
| MB 10x | -0.2114 | No | 0.2782 | 0.6183 |
| DOXY 5 µM | 0.1008 | No | 0.3415 | 0.8121 |
| DOXY 1 µM | 0.2199 | No | 0.2782 | 0.6043 |
| ATQ 100x | 0.009156 | No | 0.3715 | 0.9828 |
| ATQ 10x | 0.09041 | No | 0.3415 | 0.8312 |
| CHX 177 µM | 1.856 | Yes | <0.0001 | <0.0001 |
| PD 100x | 1.413 | Yes | 0.0008 | 0.0010 |
| PD 10x | 0.02024 | No | 0.3715 | 0.9619 |
| SBI 100x | 1.260 | Yes | 0.0021 | 0.0034 |
| SBI 10x | 1.761 | Yes | <0.0001 | <0.0001 |

**Table S7: Two-Way ANOVA analysis of parasite size for concurrent control and treatment of NF54*attB*^[ATeam1.03YEMK]^ and NF54*attB*^[sfpHluorin]^ parasites after 4 h incubation time with two-stage linear step-up procedure.**

| **Treatment** | **Mean Diff.** | **Discovery?** | **q value** | **Individual P Value** |
| --- | --- | --- | --- | --- |
|  |  |  |  |  |
| CQ 100x | 1.470 | Yes | 0.0005 | 0.0003 |
| CQ 10x | 0.3620 | No | 0.2945 | 0.3667 |
| AQ 100x | 1.697 | Yes | <0.0001 | <0.0001 |
| AQ 10x | 0.3126 | No | 0.3302 | 0.4355 |
| PYRO 100x | 1.993 | Yes | <0.0001 | <0.0001 |
| PYRO 10x | 0.1888 | No | 0.4351 | 0.6375 |
| QN 100x | 2.475 | Yes | <0.0001 | <0.0001 |
| QN 10x | 1.266 | Yes | 0.0025 | 0.0018 |
| MQ 100x | 1.614 | Yes | 0.0002 | <0.0001 |
| MQ 10x | 1.688 | Yes | <0.0001 | <0.0001 |
| LUM 100x | 0.6219 | No | 0.1133 | 0.1218 |
| LUM 10x | -0.02293 | No | 0.5211 | 0.9544 |
| DHA 100x | 2.030 | Yes | <0.0001 | <0.0001 |
| DHA 10x | 1.449 | Yes | 0.0006 | 0.0004 |
| MB 100x | -0.2095 | No | 0.4319 | 0.6012 |
| MB 10x | 0.1049 | No | 0.4513 | 0.7935 |
| DOXY 5 µM | -0.1427 | No | 0.4513 | 0.7216 |
| DOXY 1 µM | 0.1050 | No | 0.4513 | 0.7932 |
| ATQ 100x | -0.1195 | No | 0.4513 | 0.7656 |
| ATQ 10x | -0.4707 | No | 0.2055 | 0.2409 |
| CHX 177 µM | 1.770 | Yes | <0.0001 | <0.0001 |
| PD 100x | 1.182 | Yes | 0.0044 | 0.0035 |
| PD 10x | 0.6174 | No | 0.1133 | 0.1245 |
| SBI 100x | 0.7174 | No | 0.0784 | 0.0746 |
| SBI 10x | 1.110 | Yes | 0.0070 | 0.0061 |

**Table S8: pH values of compound interventions after 6 h incubation.**

| **6 h Incubation** | | **95 % Confidence Interval** | |
| --- | --- | --- | --- |
| **Compound fold EC_50_** | **Mean pH** | **Lower Limit** | **Upper Limit** |
| CQ 100x | 6.95 | 6.90 | 6.99 |
| CQ 10x | 7.35 | 7.30 | 7.40 |
| AQ 100x | 7.02 | 6.98 | 7.07 |
| AQ 10x | 7.18 | 7.13 | 7.22 |
| PYRO 100x | 6.86 | 6.82 | 6.90 |
| PYRO 10x | 7.15 | 7.11 | 7.20 |
| QN 100x | 7.17 | 7.13 | 7.22 |
| QN 10x | 7.26 | 7.21 | 7.31 |
| MQ 100x | 7.23 | 7.18 | 7.27 |
| MQ 10x | 7.21 | 7.17 | 7.26 |
| LUM 100x | 7.22 | 7.18 | 7.27 |
| LUM 10x | 7.24 | 7.19 | 7.29 |
| DHA 100x | 7.08 | 7.04 | 7.13 |
| DHA 10x | 7.23 | 7.19 | 7.28 |
| MB 100x | 7.17 | 7.12 | 7.22 |
| MB 10x | 7.33 | 7.28 | 7.38 |
| DOXY 5 µM | 7.68 | 7.62 | 7.75 |
| DOXY 1 µM | 7.38 | 7.32 | 7.43 |
| ATQ 100x | 7.23 | 7.18 | 7.28 |
| ATQ 10x | 7.29 | 7.24 | 7.34 |
| CHX 50 µg/mL | 7.23 | 7.19 | 7.28 |
| PD 100x | 7.05 | 7.01 | 7.09 |
| PD 10x | 7.30 | 7.25 | 7.35 |
| SBI 100x | 7.18 | 7.13 | 7.22 |
| SBI 10x | 7.22 | 7.18 | 7.27 |
| - GLC | 6.71 | 6.67 | 6.76 |

**Table S9: pH values of compound interventions after 4 h incubation.**

| **4 h Incubation** | | **95 % Confidence Interval** | |
| --- | --- | --- | --- |
| **Compound fold EC_50_** | **Mean pH** | **Lower Limit** | **Upper Limit** |
| CQ 100x | 6.98 | 6.94 | 7.03 |
| CQ 10x | 7.29 | 7.24 | 7.35 |
| AQ 100x | 7.11 | 7.06 | 7.15 |
| AQ 10x | 7.23 | 7.18 | 7.28 |
| PYRO 100x | 6.94 | 6.90 | 6.98 |
| PYRO 10x | 7.22 | 7.17 | 7.26 |
| QN 100x | 7.24 | 7.19 | 7.29 |
| QN 10x | 7.25 | 7.21 | 7.30 |
| MQ 100x | 7.24 | 7.19 | 7.29 |
| MQ 10x | 7.24 | 7.20 | 7.29 |
| LUM 100x | 7.26 | 7.21 | 7.31 |
| LUM 10x | 7.29 | 7.25 | 7.35 |
| DHA 100x | 7.11 | 7.07 | 7.16 |
| DHA 10x | 7.29 | 7.24 | 7.34 |
| MB 100x | 7.15 | 7.11 | 7.20 |
| MB 10x | 7.25 | 7.21 | 7.30 |
| DOXY 5 µM | 7.70 | 7.63 | 7.76 |
| DOXY 1 µM | 7.34 | 7.29 | 7.39 |
| ATQ 100x | 7.24 | 7.19 | 7.29 |
| ATQ 10x | 7.26 | 7.21 | 7.31 |
| CHX 50 µg/mL | 7.25 | 7.20 | 7.30 |
| PD 100x | 7.13 | 7.09 | 7.18 |
| PD 10x | 7.33 | 7.28 | 7.39 |
| SBI 100x | 7.17 | 7.12 | 7.22 |
| SBI 10x | 7.23 | 7.18 | 7.28 |
| - GLC | 6.70 | 6.65 | 6.75 |

**Table S10: Primer Sequences.**

| **Stable Integration** | |
| --- | --- |
| cg6F | 5’-GAAAATATTATTACAAAGGGTGAGG-3’ |
| bsdR | 5’‑ACGAATTCTTAGCTAATTCGCTTGTAAGA-3’ |
| **Molecular Cloning** | |
| ATeamF | 5’‑ATATGGATCCCCTAGGATGGTGAGCAAGGGCGAGGAGCTGT-3’ |
| ATeamR | 5’‑ATATAAGCTTCTCGAGTTACTCGATGTTGTGGCGGATCTTGAAGTTGGCCTTG-3’ |
| sfpHluprinF | 5’-ATATGGATCCCCTAGGATGAGCAAAGGAGAAGAACTTTTCAC-3’ |
| sfpHluprinR | 5’-ATATAAGCTTCCCGGGTTATTTGTAGAGCTCATCCATGCC-3’ |

References

1. Schuh AK, Rahbari M, Heimsch KC, Mohring F, Gabryszewski SJ, Weder S, Buchholz K, Rahlfs S, Fidock DA, Becker K. 2018. Stable Integration and Comparison of hGrx1-roGFP2 and sfroGFP2 Redox Probes in the Malaria Parasite Plasmodium falciparum. ACS Infect Dis 4:1601–12. doi:10.1021/acsinfecdis.8b00140

2. Nardella F, Halby L, Hammam E, Erdmann D, Cadet-Daniel V, Peronet R, Ménard D, Witkowski B, Mecheri S, Scherf A, Arimondo PB. 2020. DNA Methylation Bisubstrate Inhibitors Are Fast-Acting Drugs Active against Artemisinin-Resistant Plasmodium falciparum Parasites. ACS Cent Sci 6:16–21. doi:10.1021/acscentsci.9b00874

3. Pascual A, Henry M, Briolant S, Charras S, Baret E, Amalvict R, Des Huyghues Etages E, Feraud M, Rogier C, Pradines B. 2011. In vitro activity of Proveblue (methylene blue) on Plasmodium falciparum strains resistant to standard antimalarial drugs. Antimicrob Agents Chemother 55:2472–4. doi:10.1128/AAC.01466-10

4. Cichocki BA, Donzel M, Heimsch KC, Lesanavičius M, Feng L, Montagut EJ, Becker K, Aliverti A, Elhabiri M, Čėnas N, Davioud-Charvet E. 2021. Plasmodium falciparum Ferredoxin-NADP+ Reductase-Catalyzed Redox Cycling of Plasmodione Generates Both Predicted Key Drug Metabolites: Implication for Antimalarial Drug Development. ACS Infect Dis 7:1996–2012. doi:10.1021/acsinfecdis.1c00054

5. Berneburg I, Peddibhotla S, Heimsch KC, Haeussler K, Maloney P, Gosalia P, Preuss J, Rahbari M, Skorokhod O, Valente E, Ulliers D, Simula LF, Buchholz K, Hedrick MP, Hershberger P, Chung TDY, Jackson MR, Schwarzer E, Rahlfs S, Bode L, Becker K, Pinkerton AB. 2022. An Optimized Dihydrodibenzothiazepine Lead Compound (SBI-0797750) as a Potent and Selective Inhibitor of Plasmodium falciparum and P. vivax Glucose 6-Phosphate Dehydrogenase 6-Phosphogluconolactonase. Antimicrob Agents Chemother 66:e0210921. doi:10.1128/aac.02109-21
